# A Systematic Review of Surgical Outcomes: Comparing Robotic-Assisted Partial Nephrectomy and Open Partial Nephrectomy in Nephron-Sparing Surgery for Renal Tumors

**DOI:** 10.7759/cureus.79827

**Published:** 2025-02-28

**Authors:** Roopa Chalasani, Gilles Van de Vel, Pranav S Shukla, Shahab Ud Din Zia, Sojeong Mun, Iana Malasevskaia

**Affiliations:** 1 Research, Wake Forest Institute for Regenerative Medicine, Winston Salem, USA; 2 Hospital Medicine, King's Mill Hospital, Mansfield, GBR; 3 Medicine, Grant Medical College and Sir Jamshedjee Jeejeebhoy (JJ) Group of Hospitals, Mumbai, IND; 4 Medicine and Surgery, Pak International Medical College, Peshawar, PAK; 5 Physical Medicine and Rehabilitation, Hallym University College of Medicine, Chuncheon, KOR; 6 Obstetrics and Gynecology, Private Clinic "Yana Alexandr", Sana'a, YEM

**Keywords:** open partial nephrectomy, opn, rapn, renal cell carcinoma, renal tumors, robot-assisted partial nephrectomy

## Abstract

A partial nephrectomy is a critical approach for nephron-sparing surgery in renal tumors, striking a balance between oncological control and the preservation of renal function. Surgeons traditionally view open partial nephrectomy (OPN) as the gold standard in kidney surgery. The emergence of robotic-assisted partial nephrectomy (RAPN) offers a minimally invasive alternative that can improve surgical precision and decrease the risk of complications during the perioperative period. This systematic review analyzes and compares OPN and RAPN based on factors such as oncological effectiveness, preservation of kidney function, perioperative outcomes, and complication rates. We followed the 2020 Preferred Reporting Items for Systematic Reviews and Meta-Analyses (PRISMA) guidelines to review and include studies, analyzing 30 studies that involved more than 26,826 patients. The research team carried out retrospective and prospective cohort studies and randomized controlled trials (RCTs). They measured the quality of these studies by using the Newcastle-Ottawa Scale (NOS) for observational studies and the Cochrane Risk of Bias tool version 2 (RoB 2; Cochrane, London, UK) for RCTs. Our findings indicate that RAPN offers significant perioperative advantages, such as reduced mean estimated blood loss (EBL) of 181 mL compared to 284 mL for OPN and shorter hospital stays averaging three days compared to six days for OPN. Follow-up durations ranged from three to 60 months. Although the surgery for RAPN takes longer, it consistently preserves renal function better, resulting in less considerable declines in the estimated glomerular filtration rate (eGFR). Oncological outcomes show that RAPN achieves a negative surgical margin (NSM) rate of 97.16%, while OPN reaches 92%. RAPN effectively handles complex renal tumors, especially those with high Preoperative Aspects and Dimensions Used for Anatomical (PADUA) or R.E.N.A.L. Nephrometry score (Radius (R), Exophytic/Endophytic properties (E), Nearness to the collecting system or sinus (N), Anterior/Posterior location (A), Location relative to polar lines (L)).

In conclusion, RAPN offers significant advantages in perioperative and functional outcomes while maintaining oncological equivalence to OPN. This seals RAPN as the preferred approach in centers with robotic expertise. However, OPN remains a viable option in low-resource settings. Future research should improve access to robotic systems, standardize reporting metrics, and conduct long-term randomized trials to understand both techniques' strengths and limitations better.

## Introduction and background

Kidney cancer ranks among the 10 most common cancers in the United States, accounting for about 4% to 5% of all cases [1]. The American Cancer Society estimated that in 2024, healthcare professionals would diagnose approximately 81,610 new cases of kidney cancer. This figure included about 52,380 men and 29,230 women. They also projected around 14,390 deaths from the disease, consisting of 9,450 men and 4,940 women [1]. Most diagnoses occur in individuals aged 65, with a higher prevalence in men and among African American, American Indian, and Alaska Native populations [1]. Men have a lifetime risk of developing kidney cancer of about one in 43 (2.3%), while women face a risk of one in 73 (1.4%). Although the rate of new cases has risen, likely because of better imaging techniques, the mortality rates have dropped, showing that treatments and detection methods have improved [1].

Renal cell carcinoma (RCC) is the most frequent form of kidney cancer, making up about 85% of all renal cancer cases. It arises from the epithelium of the renal tubules and is well-recognized for its heterogeneous subtypes with different biological behaviours [2]. The incidence rates have increased significantly in recent years, especially in industrialized countries, and are primarily discovered incidentally on imaging. Treatment outcome is closely related to the stage of diagnosis, with metastatic disease having an exceptionally low five-year survival rate [2]. Surgical resection remains the primary treatment for localized RCC, with options including radical and partial nephrectomy. Radical nephrectomy (RN) involves removing the kidney along with adjacent tissues, while partial nephrectomy focuses on removing only the tumor to help maintain kidney function [3]. Surgeons commonly perform partial nephrectomy to treat RCC, particularly for small tumors or when it is essential to preserve kidney function. Initially performed via an open approach, advancements have incorporated laparoscopic methods and, more recently, robotic-assisted techniques [4]. The evolution of surgical techniques has progressed from open RN to open partial nephrectomy (OPN) and further to minimally invasive approaches, including laparoscopic partial nephrectomy (LPN) and robotic partial nephrectomy (RPN) [5]. Urology has consistently been at the forefront of adopting innovative technologies, with robotic surgery exemplifying this trend. In the United States, robotic-assisted partial nephrectomy (RAPN) has emerged as the primary method for treating renal cancer [6]. Following this trend, use of RAPN for renal masses and RCC has grown significantly in recent years [6].

Recent studies have demonstrated the effectiveness of RAPN for managing more challenging renal tumors, including those with a R.E.N.A.L. Nephrometry scores (Radius (R), Exophytic/Endophytic properties (E), Nearness to the collecting system or sinus (N), Anterior/Posterior location (A), Location relative to polar lines (L)) exceeding seven and hilar masses [5]. The RENAL score is a system designed to evaluate renal tumors based on several factors, including size, extrarenal extension, the number of tumors, anatomical anomalies, and their location within the kidney [7]. The scores range from as low as 4 to as high as 12, with higher scores advising potential surgical challenges, making it a vital factor in determining the proper surgical approach [7]. OPN has traditionally been regarded as the standard treatment for localized renal tumors classified as T1, which refers to tumors confined to the kidney and measuring 7 cm or less. However, OPN is associated with higher rates of perioperative complications and more extended hospital stays compared to minimally invasive techniques [8]. Minimally invasive approaches, like laparoscopic and robotic surgery, are becoming increasingly popular, possibly due to the invasiveness associated with traditional open surgery. RAPN demonstrates several advantages compared to other techniques, including less postoperative pain, reduced blood loss, shorter convalescence, and improved quality of life for the patient [9].

Even with the growing use of RAPN, the results of OPN have not yet been fully reviewed. The purpose of this study is to provide a comprehensive analysis of RAPN versus OPN in adults aged 18 years and older, considering renal tumors. Bridging this gap is relevant in order to provide a better understanding of the efficacy and safety of these techniques as well as making more informed and holistic clinical decisions within urology.

## Review

Methods

Study Design

This systematic review followed the Preferred Reporting Items for Systematic Reviews and Meta-Analyses (PRISMA) 2020 guidelines [10] to evaluate how RAPN compared to OPN in adults aged 18 years and older who underwent surgery for renal tumors. The primary research question guiding this review was: What were the comparative outcomes of RAPN versus OPN for these patients?

Eligibility Criteria

To be eligible for inclusion, studies focused on adults aged 18 years and older who underwent either RAPN or OPN for renal tumors. These studies reported relevant outcomes associated with RAPN or OPN and provided data on key factors such as success rates, complication rates, operative time, blood loss, recovery time, oncological outcomes, quality of life, and economic considerations. We only considered publications that were in English. Researchers utilized a variety of eligible study designs, including randomized controlled trials (RCTs), controlled clinical trials (CCTs), observational studies (such as cohort, case-control, and cross-sectional studies), comparative studies, equivalence trials, and pragmatic clinical trials.

We applied several exclusion criteria to ensure the relevance and quality of the included studies. We excluded studies involving patients younger than 18 or based on animal research. We excluded studies that focused solely on RN or other surgical techniques without reporting on RAPN or OPN. We also excluded studies that did not report relevant outcomes or focused on unrelated surgical outcomes. Furthermore, we did not include non-English publications, reviews, case series, case reports, incomplete studies, editorials, protocols, and studies without published results.

Information Sources

A comprehensive search strategy was implemented from November 11 to November 27, 2024, across multiple databases, including PubMed/MEDLINE, Cochrane Library, Science Direct, Europe PMC, ClinicalTrials.gov, and EBSCO open dissertations. The search will be based on the key concepts and Medical Subject Heading (MeSH) terms related to RAPN and OPN, renal tumor, and RCC, combined using Boolean operations in a way that allows a comprehensive retrieval of studies relevant for this paper.

Search Strategy

The search strategy involved several key concepts and MeSH terms. The first concept focused on “Robotic-assisted partial nephrectomy” OR “RAPN”, the second concept focused on “Open partial nephrectomy” OR “OPN”, and the third concept included “Renal tumor” OR “Renal cell carcinoma”. We used MeSH terms such as “Robotic Surgical Procedures/adverse effects”, “Robotic Surgical Procedures/mortality”, “Robotic Surgical Procedures/statistics and numerical data”, “Nephrectomy/adverse effects”, “Nephrectomy/mortality”, “Nephrectomy/statistics and numerical data”, “Carcinoma, Renal Cell/surgery”, “Carcinoma, Renal Cell/prevention and control”, “Carcinoma, Renal Cell/mortality”. We integrated these concepts to create a thorough search that captures all relevant studies (Table 1).

**Table 1:** Search strategy I/E: Inclusion/exclusion; MEDLINE: Medical Literature Analysis and Retrieval System Online; CENTRAL: Cochrane Central Register of Controlled Trials; PMC: PubMed Central; EBSCO: Elton B. Stephens Company.

Search strategy	Databases	Number of papers identified before applying I/E criteria	Filters applied	Number of papers identified after applying I/E criteria
(("Robotic-assisted partial nephrectomy" OR "RAPN" OR ((((("Robotic Surgical Procedures/adverse effects"[Mesh]) OR ( "Robotic Surgical Procedures/mortality"[Mesh] )) OR ( "Robotic Surgical Procedures/statistics and numerical data"[Mesh] )) AND "Nephrectomy/adverse effects"[Mesh]) OR ( "Nephrectomy/mortality"[Mesh] )) OR ( "Nephrectomy/statistics and numerical data"[Mesh] )) OR ("Open partial nephrectomy" OR "OPN")) AND ((("Carcinoma, Renal Cell/surgery"[Mesh]) OR ( "Carcinoma, Renal Cell/prevention and control"[Mesh] OR )) OR ( "Carcinoma, Renal Cell/mortality"[Mesh]))	PubMed/MEDLINE	666	Clinical trial, comparative study, controlled clinical trial, equivalence trial, observational study, pragmatic clinical trial, randomized controlled trial, English publications.	165
#1 "robotic assisted partial nephrectomy" OR "robotic partial nephrectomy" OR "robot-assisted nephrectomy" OR "robotic-assisted nephrectomy" OR "RAPN" 149 #2 "open partial nephrectomy" OR "traditional partial nephrectomy" OR "conventional partial nephrectomy" OR "open nephrectomy" 137 #3 "renal cell carcinoma" OR "kidney cancer" OR "renal carcinoma" OR "renal neoplasm" OR "clear cell carcinoma" 3865 #4 MeSH descriptor: [Carcinoma, Renal Cell] explode all trees 1568 #5 #3 OR #4 4087 #6 #1 OR #2 AND #5 176	Cochrane Library (CENTRAL)	176	Clinical trials, English publications.	172
(“Robotic-assisted partial nephrectomy” OR “RAPN” OR “Open partial nephrectomy” AND “Renal tumor” OR “Renal cell carcinoma”) AND (HAS_FT:Y OR (HAS_FREE_FULLTEXT:Y))	Europe PMC	1,164	Full text in Europe PMC, link to free full text, research articles.	786
“Robotic-assisted partial nephrectomy” OR “RAPN” OR “Open partial nephrectomy” AND “Renal tumor” OR “Renal cell carcinoma”	ClinicalTrials.gov	26	Interventional, observational studies, completed, studies with results, age- 18+years	2
(“Robotic-assisted partial nephrectomy” OR “RAPN” OR “Open partial nephrectomy”) AND (“Renal tumor” OR “Renal cell carcinoma”)	Science Direct	1,828	Title, abstract, keywords: Robotic assisted partial nephrectomy, open partial nephrectomy, renal tumors, renal cell carcinoma, English publications.	21
(“Robotic-assisted partial nephrectomy” OR “RAPN” OR “Open partial nephrectomy” ) AND (“Renal tumor” OR “Renal cell carcinoma”) AND ( “cohort” OR ''cross sectional study'' OR '' observational study'' OR ''case control'' OR “clinical trials” OR “controlled clinical trial” OR “randomized clinical trial” OR “non randomized clinical trial” ) NOT (''Review'' OR ''Meta-analysis'' OR ''poster' OR "commentary")	EBSCO Open Dissertations	181	Cross-sectional, cohort, observational and case control studies, clinical trials, controlled clinical trials, randomized clinical trial or non-randomized clinical trial.	26

Selection and Short-Listing Process

Our research team used the Rayyan app (Cambridge, MA) to conduct the screening process of the studies [11]. We excluded duplicates and irrelevant studies after screening the titles and abstracts of the studies. Following this, we extracted the data from remaining studies and performed full-text screening on those that met our eligibility criteria. Finally, we compiled the studies that satisfied our inclusion criteria.

Quality Appraisal Based on the Design of Included Studies

We evaluated the potential for bias in the studies using appropriate tools based on each study design. We assessed the risk of bias for RCTs with the Cochrane Risk of Bias Tool (RoB 2; Cochrane, London, UK) [12]. For observational studies, we assessed quality using the Newcastle-Ottawa Scale (NOS) [13]. Next, we evaluated the methodological rigor and reliability of the study findings.

Data Synthesis

Researchers interpreted the data descriptively and presented it in narrative and tabular form to compare outcomes from RAPN and OPN. The table demonstrates the main results from the studies on success rates, complications, recovery time, oncology response, quality of life, and cost, highlighting the benefit of RAPN over OPN. The table also shows individual results, allowing for comparisons that help visualize the data and illustrate the full scope of the advantages and disadvantages of the surgery.

Results

Study Selection

**Figure 1:**
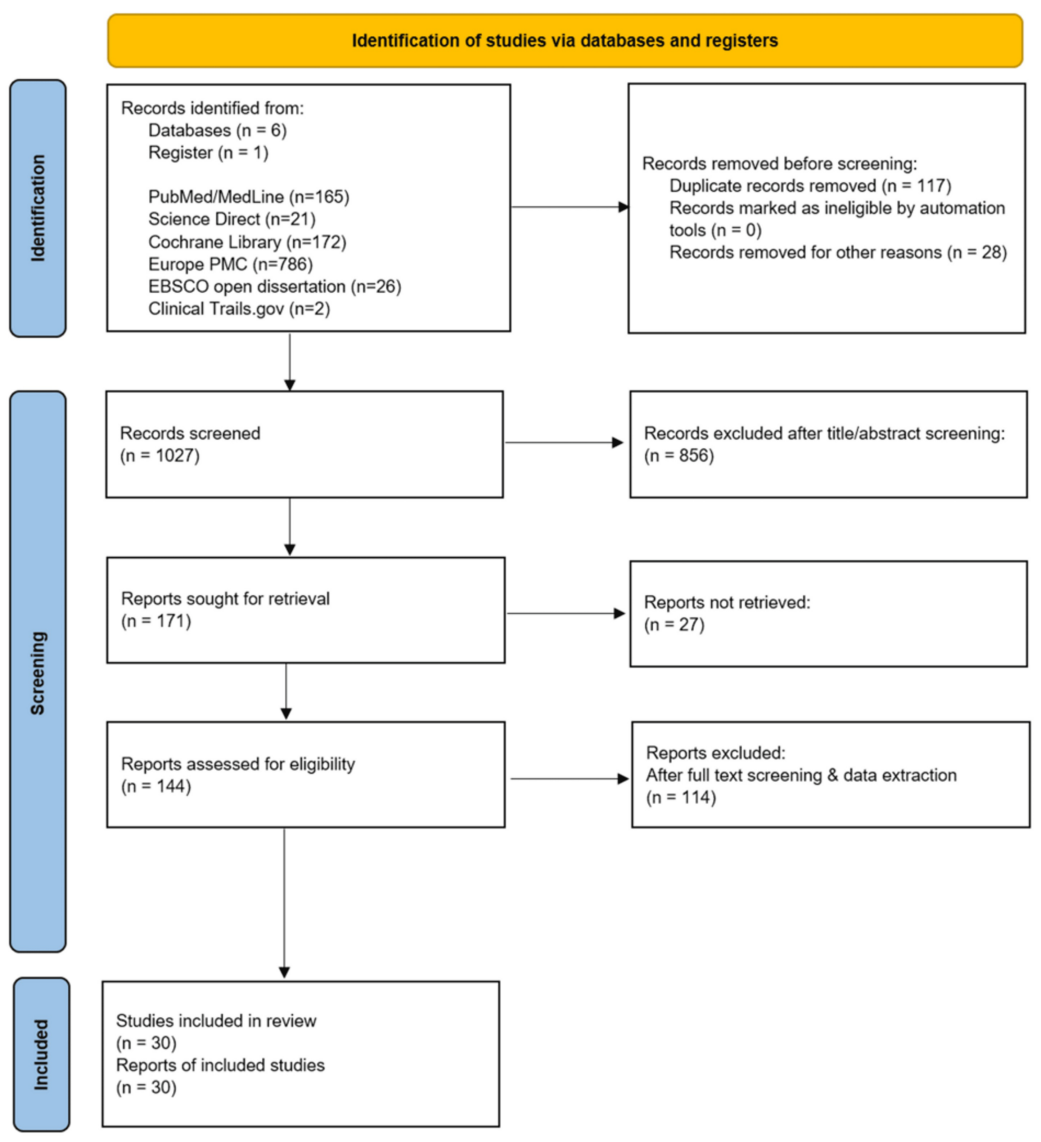
PRISMA 2020 diagram PRISMA: Preferred Reporting Items for Systematic Reviews and Meta-Analyses; PubMed Central; MEDLINE: Medical Literature Analysis and Retrieval System Online; EBSCO: Elton B. Stephens Company.

A summary of the study selection using the PRISMA flow diagram can be found in Figure 1.

Risk of Bias Assessment

The included studies used appropriate tools to assess the risk of bias. For RCTs, researchers employed the Cochrane risk of bias tool (RoB 2) to evaluate potential biases across five domains: selection bias, deviations from intended interventions, missing outcome data, measurement of outcomes, and selection of reported results [12]. Researchers utilized the NOS for observational studies to assess study quality, focusing on selection, comparability, and outcome assessment [13].

In this systematic review, we included only studies classified as having “some concerns” or “low risk of bias” according to the Cochrane RoB 2 tool [12], along with observational studies that achieved a minimum score of 7 out of 9 on the NOS scale assessment [13]. This structured methodology allowed us to rigorously evaluate the comparative outcomes of RAPN and OPN, providing valuable insights into surgical practices for renal tumors. Table 2 summarizes the risk of bias for 29 observational studies evaluated by the NOS [13].

**Table 2:** Quality appraisal using Newcastle Ottawa scale Selection (maximum of 4 stars); Comparability (maximum of 2 stars); Outcome (maximum of 3 stars); Overall good quality: 7-9 stars

Authors	Study design	Selection	Comparability	Outcome	Total score	Comments
Love et al. [4]	Retrospective cohort study	****	**	***	9	Good quality
Wang et al. [5]	Retrospective cohort study	****	**	***	9	Good quality
Xia et al. [6]	Retrospective cohort study	****	**	***	9	Good quality
Roaldsen et al. [8]	Retrospective cohort study	****	**	***	9	Good quality
Lee et al. [9]	Retrospective cohort study	****	**	***	9	Good quality
Yamanoi et al. [14]	Retrospective cohort study	****	**	***	9	Good quality
Jang et al. [15]	Retrospective cohort study	****	*	***	8	Good quality
Mahmud et al. [16]	Retrospective cohort study	****	**	***	9	Good quality
Chu et al. [17]	Retrospective cohort study	****	**	***	9	Good quality
Mastroianni et al. [18]	Retrospective cohort study	****	**	***	9	Good quality
Kumar et al. [19]	Retrospective cohort study	****	**	***	9	Good quality
Prata et al. [20]	Prospective cohort study	****	**	**	8	Good quality
Masoumi-Rvandi et al. [21]	Retrospective cohort study	****	**	***	9	Good quality
Kawase et al. [22]	Retrospective multicenter cohort study	****	*	***	8	Good quality
Soisrithong et al. [23]	Retrospective cohort study	****	**	***	9	Good quality
Takahara et al. [24]	Retrospective cohort study	****	*	***	8	Good quality
Lyu et al. [25]	Retrospective cohort study	****	*	***	8	Good quality
Yu et al. [26]	Retrospective cohort study	****	*	***	8	Good quality
Xu et al. [27]	Retrospective cohort study	****	*	***	8	Good quality
Seyam et al. [28]	Retrospective cohort study	****	*	***	8	Good quality
Boylu et al. [29]	Prospective cohort study	****	*	**	7	Good quality
Kim et al. [30]	Retrospective cohort study	****	*	***	8	Good quality
Zapala et al. [31]	Case control	****	**	***	9	Good quality
Xu et al. [32]	Retrospective cohort study	****	**	***	9	Good quality
Wu et al. [33]	Retrospective cohort study	****	**	***	9	Good quality
Oh et al. [34]	Retrospective cohort study	****	**	***	9	Good quality
Hankins et al. [35]	Retrospective cohort study	****	**	***	9	Good quality
Abdullah et al. [36]	Multicenter retrospective cohort study	****	**	***	9	Good quality
Buffi et al. [37]	Multicenter retrospective cohort study	****	**	***	9	Good quality

Table 3 summarizes the risk of bias for one RCT study evaluated by Cochrane risk of bias RoB 2 [12]. The ROBOCOP II trial by Abate et al. [38] presents concerns primarily about the open-label design and handling of missing data. Future studies should aim for larger sample sizes and consider blinding to minimize bias further.

**Table 3:** Cochrane risk of bias 2 assessment of randomized studies D=domain; D 1) Selection bias, outcome; D 2) Deviations from intended interventions; D 3) Missing outcome data; D 4) Measurement of outcome; D 5) Selection of reported result.

Study	D1	D2	D3	D4	D5	Overall
Abate et al. [38]	Low risk	Some concerns	Some concerns	Low risk	Low risk	Some concerns

Summary of Included Studies

The studies included are summarized in Table 4 and consist of 30 studies comparing RAPN and OPN, totaling 26,826 patients. Most studies are retrospective and prospective cohort studies, with only one RCT, these offer high-quality data on perioperative, oncologic, and functional outcomes. Sample sizes ranged from single-center studies to large multicenter cohorts, including a range of 50-1,836 patients. The outcomes assessed included perioperative metrics such as estimated blood loss (EBL), operative time, length of hospital stay, oncologic efficacy regarding recurrence-free survival and favorable surgical margin rates, functional outcomes reflecting estimated glomerular filtration rate (eGFR) preservation, and complication profiles. Follow-up periods ranged from three months to more than 60 months. The Clavien-Dindo classification reported complications, showing that OPN had higher rates of severe complications, such as perioperative bleeding, wound infections, and vascular events. In comparison, RAPN had lower complication rates and quickened recovery. These series collectively show the benefits of RAPN in perioperative outcomes while proving that it is similarly effective in oncologic and functional aspects as OPN. However, the studies' retrospective nature and variation in follow-up periods indicate a need for standardized prospective trials. Table 4 summarizes procedures, patient details and reported outcomes of the studies.

**Table 4:** Outcomes of included studies RAPN: Robotic-assisted partial nephrectomy, OPN: Open partial nephrectomy, N: Total number of patients, PSM: Positive surgical margin, NSM: Negative surgical margin, WIT: Warm ischemia time, EBL: Estimated blood loss, QoL: Quality of life, eGFR: Estimated glomerular filtration rate, LPN: Laparoscopic Partial Nephrectomy, RALPN: Robotic-assisted laparoscopic partial nephrectomy, IG-PCA: Image-guided percutaneous cryoablation,  PLOS: Prolonged length of hospital stay, RCC: Renal cell carcinoma, OS: Overall survival rate, DFS: Disease free survival rate, CSS: Cancer specific survival rate, RFS: Recurrence free survival rate, RENAL Nephrectomy score: (Radius (R), Exophytic/Endophytic properties (E), Nearness to the collecting system or sinus (N), Anterior/Posterior location (A), Location relative to polar lines (L)), PADUA score: Preoperative Aspects and Dimensions Used for an Anatomical score, RMxPNx: Robotic-assisted multiport partial nephrectomy, Off-C: off-clamp, NCDB: National Cancer Database, ICU: Intensive care unit, IQR: Interquartile range, LRFS: Local recurrence-free survival, MFS: Metastasis-free survival, CKD: Chronic kidney disease, T1a: Tumor is ≤4 cm, T1b: Tumor is >4 cm but ≤7 cm.

Study	Procedure	Patient details	Reported outcomes
Love et al. [4]	OPN vs. RAPN	N=1,234 patients. OPN:385. RAPN:849. mean age 58.8 years. Median RENAL score: 7+/- 1.8	RAPN: Ischemia time: 16 minutes (post-2012 improvements), 5-year CSS: 98.7%, 5-year OS: 93.5%, RFS: Better than OPN, Serum creatinine: Stable at follow-up, less increase compared to OPN. OPN: Ischemia time: 19 minutes (post-2012 improvements), 5-year CSS: 96.4%, 5-year OS: 90.2%, RFS: Lower than RAPN, Serum creatinine: Stable at follow-up, slight increase compared to baseline.
Wang et al. [5]	RAPN vs. OPN	N=380 patients. RAPN: 190. OPN: 190. Mean age: RAPN: 61.8 years (SD = 12.3). OPN: 59.8 years (SD = 11.8). Median tumor size: 4.2 cm. RENAL scores ≥7; matched for age, sex, BMI, and tumor complexity.	RAPN: Shorter hospital stay: 7.8 days (vs. 9.2 days for OPN), EBL: 196.8 mL (vs. 240.8 mL for OPN), WIT: 21.3 minutes (vs. 23.3 minutes for OPN), PSS: 3.1% (comparable to OPN at 2.6%), 5-year CSS: 98.7%, 5-year RFS: 95.1%. OPN: hospital stay: 9.2 days, higher blood loss: 240.8 mL, WIT: 23.3 minutes, PSS: 2.6% (comparable to RAPN), 5-year CSS: 96.4%, 5-year RFS: 92.7%.
Xia et al. [6]	RAPN data from the NCDB (2010–2013).	N=18,724 patients with RCC treated with RAPN. Mean age: 59.4 years, predominantly male. Majority had a low comorbidity burden (Charlson-Deyo Index of 0).	Mortality: 30-day: 0.26%; 90-day: 0.41%, Conversion to Open Surgery: Higher rates in Very Low Volume Hospitals. Odds of conversion significantly reduced at Very High Volume Hospitals (OR: 0.47, P < 0.001). PLOS: Very High Volume Hospitals: Odds of PLOS were 55% lower compared to Very Low Volume Hospitals (OR: 0.45, P < 0.001). PSM: Very Low Volume Hospitals: 11.8%. Very High Volume Hospitals: 4.6%. Higher hospital volume correlated with significantly reduced odds of PSM (OR: 0.34, P < 0.001).
Roaldsen et al. [8]	OPN, RAPN	N=197 patients with suspected RCC. OPN: 75 patients. RAPN: 122 patients. Mean age: 63 years (OPN), 62 years (RAPN). Mean RENAL nephrometry score: 6.6 (OPN), 6.9 (RAPN).	Operative Time: OPN shorter (81 min) vs. RAPN (144.5 min; P < 0.001). Blood Loss: OPN higher (227 mL) vs. RAPN (189 mL; P = 0.1). Hospital Stay: OPN longer (6 days) vs. RAPN (3 days; P < 0.001). PSM: OPN higher (21.6%) vs. RAPN (4.2%; P < 0.001). Renal Function (eGFR): eGFR (Postoperative):OPN: 72 ± 22 mL/min/1.73 m². RAPN: 70 ± 20 mL/min/1.73 m². No significant difference between the groups postoperatively (P = 0.7). Change in eGFR: OPN: Mean decrease of 12 ± 11 mL/min/1.73 m². RAPN: Mean decrease of 14 ± 14 mL/min/1.73 m². No significant difference in mean eGFR decline between the groups (P = 0.3). Ischemia Time: OPN- Majority performed off-clamp (68/75 cases), with minimal renal artery ischemia. Mean ischemia time (for on-clamp cases): 10.4 ± 4 minutes (n = 7). RAPN- Renal artery clamped in all cases. Mean ischemia time: 14.6 ± 6 minutes. The longer ischemia time in RAPN did not translate to significant differences in postoperative renal function.
Lee et al. [9]	OPN, RAPN	N=134 patients with renal tumors ≥4 cm. OPN: 67 patients. RAPN: 67 patients. OPN: Mean age 52 years. RAPN: Mean age 53 years. RENAL Nephrometry Score- OPN: 7.6. RAPN: 7.8	Surgical metrics: Operative Time: OPN: 149 minutes. RAPN: 173.3 minutes (P = 0.030, longer in RAPN). Ischemia Time: OPN: 20.3 minutes. RAPN: 29.4 minutes (P = 0.001, longer in RAPN). EBL: OPN: 271.7 mL. RAPN: 198.7 mL (P = 0.053, not statistically significant). Hospital Stay: OPN: 8.2 days. RAPN: 6.0 days (P = 0.001, shorter in RAPN). eGFR Preservation: 1-month postoperative eGFR change: OPN: 8.4 mL/min/1.73 m². RAPN: 7.5 mL/min/1.73 m² (P = 0.638, no significant difference). 6-month postoperative eGFR change: OPN: 8.2 mL/min/1.73 m². RAPN: 3.1 mL/min/1.73 m² (P = 0.027, significantly better in RAPN). RFS: 5-year RFS: OPN 94.6%, RAPN 98.4% (P = 0.970, no significant difference). CSS: 5-year CSS: OPN 98.4%, RAPN 100% (P = 0.345, no significant difference). PSM: None in either group.
Yamanoi et al. [14]	RAPN, IG-PCA	N=216 patients with cT1 RCC. RAPN: 108 patients. IG-PCA: 108 patients. Mean age (years): RAPN:66 years. IG-PCA:65 years.	LRFS: RAPN: 100%. IG-PCA: 96.5% (P = 0.11, not significant). MFS: RAPN: 98.9%. IG-PCA: 97.9% (P = 0.64, not significant). OS: RAPN: 96.8%. IG-PCA: 90.2% (P = 0.17, not significant). Renal Function Preservation: No significant differences in eGFR preservation between RAPN and IG-PCA over 5 years. Greater than 90% eGFR preservation at 12 months: RAPN: 46.2%. IG-PCA: 47.5% (P = 0.87).
Jang et al. [15]	RAPN, LPN	N=127 patients with renal tumors. LPN: 38 patients RAPN: 89 patients Mean age: RAPN: 49.1 ± 12.6 years LPN: 54.7 ± 11.8 years​ Tumor Complexity (RENAL Score ≥7): LPN: 52.6% of tumors classified as high complexity. RPN: 48.4% of tumors classified as high complexity.	Operative Time: LPN:168.5 minutes. RPN:143.9 minutes (P = 0.061; not statistically significant). WIT: LPN: 27.3 minutes. RPN: 24.7 minutes (P = 0.171; not statistically significant). EBL: LPN: 156.8 mL. RPN: 197.6 mL (P = 0.142; not statistically significant). Hospital Stay: LPN: 7.2 days. RPN: 7.1 days (P = 0.090; not statistically significant). PSM: LPN: 2.6%. RPN: 0% (P = 0.299; not statistically significant). Margin Width: LPN: 0.6 cm. RPN: 0.4 cm (P = 0.001; significant). Postoperative eGFR: LPN: 69.8 mL/min/1.73 m². RPN: 77.3 mL/min/1.73 m² (P = 0.035; significant).
Mahmud et al. [16]	RAPN, LPN, OPN	N= 785 patients. RAPN: 398. OPN: 265. LPN: 122. Mean age (years ± SD): 62.2 ± 6.3. RENAL Nephrometry Score: RAPN: 7 (5–9). OPN: 8 (5–9). LPN: 7 (6–9).	Operation time: RAPN:189.3 ± 71.5 minutes OPN:198.3 ± 71.5 minutes LPN:235.2 ± 75.5 minutes. EBL (mL): RAPN:180 (50–350) OPN:230 (50–400) LPN:255 (50–350) Ischemia time (min): RAPN: 22.4 (12–32) OPN: 24.3 (12–32) LPN: 23.4 (9–33). Time to Embolization (days): RAPN: 16 (5–31) OPN: 13 (1–20) LPN: 11 (7–18). eGFR preservation (%): RAPN: 85.2 OPN: 84.3 LPN: 85.2 Clinical stage: RAPN: T1a (90.95%), T1b (9.1%) OPN: T1a (90.56%), T1b (9.4%). LPN: T1a (87.7%), T1b (12.29%). Benign pathology: RAPN: 13%. OPN: 15%. LPN: 18%.
Chu et al. [17]	OPN, RLPN.	N=130 patients. OPN= 65. RLPN=65. Mean age (years ± SD): OPN: 59.72 ± 4.21. RLPN: 60.18 ± 3.95. RENAL Nephrometry Score: RLPN= 8.69 ± 1.55. OPN= 8.74 ± 1.43.	Surgical time: RLPN: 127.22+/- 15.67 OPN: 134.52+/- 16.32 EBL: RLPN: 205.45± 38.89, OPN: 230.34 ± 55.67, WIT (min): RLPN: 24.03 ± 38.89, OPN: 19.92 ± 8.75. PSM (%): RLPN:2.48 ± 0.68, OPN: 2.35 ± 0.71. Hospital time (days): RLPN: 4.98 ± 1.65, OPN: 4.85 ± 1.32, 3- year overall survival rate (%): RLPN: 87.69%, OPN: 84.62%. 3-year disease free survival rate (%): RLPN: 83.08%, OPN: 80%. 3- year CSS rate (%): RLPN: 90.77%, OPN: 86.15%.
Mastroianni et al. [18]	Off-C RAPN, Off-C OPN	N=542 patients. Mean age (years ± SD): 61.3 ±11.9. Off-C OPN: 147. Off-C RAPN: 395.	Hospital Stay: Off-C RAPN: 3.4 ± 1.7 days Off-C OPN: 5.4 ± 1.9 days. Trifecta Achievement: Off-C RAPN: 89.8% Off-C OPN: 80.3% PSM: Off-C RAPN: None observed in Off-C RAPN (0%). Off-C OPN: Present in 6.1% of cases, reflecting challenges in achieving clean excisions with the open approach. Transfusion Rate: Off-C RAPN: Lower at 3.4%, reflecting better intraoperative hemostasis and less blood loss. Off-C OPN: 12.2%, associated with greater intraoperative blood loss. Oncological Outcomes: CSS, OS, DFS, and MFS were comparable between groups, confirming the oncological safety of Off-C RAPN.
Kumar et al. [19]	OPN, RAPN, LPN.	N=62 patients. OPN: 15. LPN: 12 RAPN: 25. OPN (mean age: 63 years), LPN and RAPN (mean ages: 58 and 57 years, respectively). RENAL Nephrometry Score: OPN: 8 ± 2 LPN: 7 ± 1.5 RAPN: 6.5 ± 1.2	OPN: Performed on the largest and most complex tumors (mean size 6.6 ± 2.3 cm). Shortest WIT (18 minutes). The highest recorded blood loss was 450 mL, which is significantly greater than that of other methods. Most extended hospital stay (6 days). PSM in 2 cases (AML). LPN: Tumor size was 3.8 ± 0.99 cm, representing intermediate complexity. WIT: 25 minutes. EBL: 150 mL, significantly lower than OPN. Hospital stay averaged 5 days. PSM in 1 case (papillary RCC). RAPN: Performed on smaller tumors (3.9 ± 1.17 cm). The most extended operation lasted 180 minutes. WIT: 25 minutes. The blood loss was minimal, measuring only 150 mL. Hospital stay averaged 5 days. No PSM observed.
Prata et al. [20]	RAPN, LPN.	N= 89 patients. RAPN: 27. LPN: 62. Mean age: RAPN: 56 years. LPN: 58 years. RENAL Nephrometry Score: RAPN: 6.5 ± 1.2. LPN: 7 ± 1.5.	RAPN: Operative Time: 92 minutes, significantly shorter than LPN (P = 0.005). Hospital Stay: 3 days, shorter than LPN (P = 0.002). PSM: 3.7%, lower than LPN (not statistically significant). Trifecta Achievement: 92.6%, higher than LPN but not statistically significant (P = 0.10). eGFR at Discharge: Comparable to LPN, indicating similar renal functional preservation. LPN: Operative Time: 149.5 minutes, significantly longer than RAPN. Hospital Stay: 5 days, longer than RAPN. PSM: 4.8%, slightly higher than RAPN. Trifecta Achievement: 82.3%, lower than RAPN but not statistically significant. eGFR at Discharge: Comparable to RAPN.
Masoumi-Rvandi et al. [21]	RALPN, OPN, LPN.	N=271 patients. RALPN: 82. LPN: 83. OPN: 106. Mean age (years): RALPN: 57.98. LPN: 52.10. OPN: 55.80. RENAL Nephrometry score: RALPN: 7.05 (median). LPN: 6.32 (median). OPN: 7.55 (median).	RALPN: Operative Time: Shortest at 82 minutes. EBL: Moderate at 165 mL. Hospital Stay: Shortest at 2 days. WIT: Longest at 17 minutes. OPN: Operative Time: Longest at 104 minutes. EBL: Highest at 250 mL. Hospital Stay: Longest at 4 days. WIT: Shortest at 14.5 minutes. LPN: Operative Time: Moderate at 96 minutes. EBL: Lowest at 125 mL. Hospital Stay: Moderate at 3 days. WIT: Moderate at 15 minutes.
Kawase et al. [22]	RAPN, LPN	N=269 patients. RAPN: 100 patients. LPN: 169 patients. Mean age: RAPN: 65 years (IQR: 55–73). LPN: 64 years (IQR: 56–72). RENAL Nephrometry score: RAPN: 6 (IQR: 5–8). LPN: 6 (IQR: 5–8).	RAPN: Operative Time: Shorter at 200 minutes (IQR: 167–240). EBL: 50 mL (IQR: 10–150), reflecting better hemostatic control. WIT: Shortest at 18 minutes (IQR: 16–23). Trifecta Achievement: Achieved in 74% of patients, significantly higher than LPN (P < 0.001). Postoperative eGFR Decline: Minimal with no significant long-term renal function loss. LPN: Operative Time: Longer at 233 minutes (IQR: 183–279). EBL: 20 mL (IQR: 10–100). WIT: Longer at 25 minutes (IQR: 19–31). Trifecta Achievement: Achieved in 39.6% of patients, significantly lower than RAPN (P < 0.001). Postoperative eGFR Decline: Slightly higher, with 7.7% of patients experiencing ≥15% eGFR reduction at 3 months.
Soisrithong et al. [23]	OPN, RAPN, LPN	N=70 patients. OPN: 18 patients. LPN: 11 patients. RAPN: 41 patients. Mean age (years): OPN: 55.5 years (± 15.26). LPN: 60.9 years (± 8.44). RAPN: 58.36 years (± 13.48). RENAL Nephrometry Score: OPN: Median 7 (range: 6–9). LPN: Median 7 (range: 6–8). RAPN: Median 7 (range: 6–9).	OPN: Shortest operative time: 135 minutes. EBL: 400 mL (IQR: 200–700). Transfusion rate: 33%. Median hospital stay: 6 days (IQR: 6–10). Trifecta achievement: 64.29%. Positive Margins: 0%. Negative Margins: 16 cases (100%) Postoperative eGFR(median at 1 year): 90 mL/min/1.73 m² (IQR: 84–103). Patients achieving ≥90% eGFR at 1 year: 43.75%. RAPN: Longest operative time: 225 minutes. EBL: 300 mL (IQR: 200–450). Median hospital stay: 6 days (IQR: 5–7)​ Transfusion rate: 7.32%. Highest trifecta achievement: 64.71%. Hospital stay: 6 days. Positive margin: 2 (4.88 %) Negative margin: 39 (95.12 %) Postoperative eGFR (median at 1 year): 94 mL/min/1.73 m² (IQR: 78–98).Patients achieving ≥90% eGFR at 1 year: 32.35%​​. LPN: Operative time: 189 minutes. EBL: 250 mL (IQR: 50–600). Median hospital stay:6 days (IQR: 5–6). Transfusion rate: 9.09%. Trifecta achievement: 45.45%. Positive margin: 0% Negative Margins:11 (100%). Postoperative eGFR (median at 1 year): 87 mL/min/1.73 m² (IQR: 79–102). Patients achieving ≥90% eGFR at 1 year: 54.55%.
Takahara et al. [24]	RAPN, OPN	N=78 patients. RAPN: 39 patients (post-matching). OPN: 39 patients (post-matching). Mean age (years) RAPN: 62 years (range: 29–85). OPN: 67 years (range: 35–78). Both RAPN and OPN had a median RENAL score of 7 (range: 4–11).	RAPN: Operative Time: Median 175 minutes (range: 103–267).EBL: Significantly lower at 50 mL (P < 0.001). Ischemia Time: Shorter at 18 minutes (P < 0.001). Postoperative eGFR: At 1 year: 61.2 mL/min. At 3 years: 55.5 mL/min. PSM: None reported in RAPN. Transfusion rate:7.7%. OPN: Operative Time: Median 169 minutes (range: 84–325). EBL: Higher at 174 mL (P < 0.001). Ischemia Time: Longer at 24 minutes. Postoperative eGFR: At 1 year: 54.8 mL/min. At 3 years: 54.8 mL/min. PSM: None reported in OPN. Transfusion rate: 7.7%.
Lyu et al. [25]	RAPN	N=896 patients Median age: 52 years (range: 14–86 years). RENAL Nephrometry Score:Median score: 7 (range: 4–11).	Surgical Time: Median 120 minutes (range: 40–385). WIT: Median 18 minutes (range: 6–40). EBL: Median 50 mL (range: 5–2000). Oncological Outcomes: PSM: 0.45%, reflecting high precision in tumor excision. Disease Progression: Recurrence: 0.9%. Metastasis: 2.1%. Death: 1.0% (median follow-up: 20.2 months). Renal Function Mean eGFR decline at 1 year: 14.6% ± 19% compared to baseline. Preservation of ≥90% eGFR at 1 year was achieved in 50.4% of tumors ≤4 cm and 29.6% of tumors >4 cm. .
Yu et al. [26]	OPN, LPN	N=210 patients. OPN: 91 patients. LPN: 119 patients. Mean age (years): OPN: 57.9 ± 13.9. LPN: 57.6 ± 9.6. RENAL Nephrometry Score: Low Complexity Group: 4–6 points. Moderate Complexity Group: 7–9 points.	OPN: Low Complexity Group: Operative Time: 115.1 minutes (mean). WIT (Clamp Time): 11.9 minutes. EBL: 137.1 mL. Hospital Stay: 8.0 days (mean). Moderate Complexity Group: Operative Time: 121.0 minutes (mean). WIT: 16.2 minutes. EBL: 181.2 mL. Hospital Stay: 8.2 days. PSM:1(2.4%). LPN: Low Complexity Group: Operative Time: 142.5 minutes (mean). WIT(Clamp Time): 17.0 minutes. EBL: 85.6 mL. Hospital Stay: 7.0 days (mean). Moderate Complexity Group: Operative Time: 154.5 minutes (mean). WIT: 23.4 minutes. EBL: 165.5 mL. Hospital Stay: 8.3 days. PSM: 2(3.8%)
Xu et al. [27]	RAPN using the KD-SR-01 robotic system	N=17 patients. Median age: 51 years (range: 36–72). RENAL Nephrometry Score: Median score: 4–6 in 12 cases (70.6%) and 7–9 in 5 cases (29.4%).	Operative Time: Median 110.5 minutes (mean: 68.6 minutes for robotic operative time). WIT: 16.9 minutes, well within safe thresholds. EBL: Median 50 mL (range: 50–200 mL). Renal Function eGFR: Preoperative: 97.9 mL/min. Postoperative Day 1: 91.7 mL/min (P = 0.036). Postoperative Day 4: 95.7 mL/min (P = 0.427, indicating recovery). Preservation: The KD-SR-01 system demonstrated effective renal function preservation comparable to other robotic platforms. Oncological Outcomes Surgical Margins: All margins were negative (100%), confirming oncological safety.
Seyam et al. [28]	RAPN	N=101 patients. Mean age (years): 47.2 years (range: 21–77). RENAL Nephrometry Score: Median score: 6 (range: 4–10).	Operative Time: Median 166 minutes (range: 66–381). WIT: Median 17 minutes. EBL: Median 200 mL (range: 5–1500). PSM: 5%. Renal Function eGFR Preservation: Median eGFR decline: 5.9%. Percentage of patients with ≥15% decline in eGFR: 15.8%.
Boylu et al. [29]	RAPN, OPN	N=66 patients. RAPN: 46 patients. OPN: 20 patients. Mean age (years): RAPN: 54 years (±12). OPN: 56 years (±13.5). RENAL Nephrometry Score RAPN: 5.35 ± 1.6 OPN: 6.35 ± 1.7 (P = 0.02).	RAPN: Operative Time: 225 ± 58 minutes, longer than OPN. EBL: 268 ± 303 mL, significantly lower than OPN. WIT: 23.3 ± 7.3 minutes, longer than OPN. Hospital Stay: 4.1 ± 1.5 days, shorter than OPN. Renal Function: Preoperative eGFR: 90.8 mL/min/1.73 m². Postoperative eGFR (3 months): 84.5 mL/min/1.73 m², not significantly different from OPN. PSM: 1 (2.1%) OPN: Operative Time: 152 ± 18 minutes, shorter than RAPN. EBL: 417 ± 202 mL, significantly higher than RAPN. WIT: 18 ± 3.5 minutes, shorter than RAPN. Hospital Stay: 5.4 ± 2 days, longer than RAPN. Renal Function: Preoperative eGFR: 88 mL/min/1.73 m². Postoperative eGFR (3 months): 79.8 mL/min/1.73 m², not significantly different from RAPN. PSM: 0%
Kim et al. [30]	RAPN, OPN	N=149 patients. OPN: 64 patients. RPN: 85 patients. Mean age (years): OPN: 52.0 years (IQR: 40.5–60.5). RPN: 53.0 years (IQR: 42.0–60.0). RENAL Nephrometry Score: Median score: OPN: 10.1 (IQR: 10.0–10.0). RAPN: 10.2 (IQR: 10.0–10.0).	RAPN: Operative Time: Median 150 minutes (IQR: 110–190). EBL: Median 200 mL (IQR: 100–300). WIT: Median 24 minutes (IQR: 19–34). Hospital Stay: Median 5 days (IQR: 5–7). Renal Function: eGFR decline: 6.5 mL/min/1.73m² from baseline at last follow-up. De novo CKD: 4.9%. PSM: 0%. OPN: Operative Time: Median 145 minutes (IQR: 105–180). EBL: Median 200 mL (IQR: 100–300). WIT: Median 21 minutes (IQR: 18–30). Hospital Stay: Median 7 days (IQR: 5–9). Renal Function: eGFR decline: 3.8 mL/min/1.73m² from baseline at last follow-up. De novo CKD: 3.3%. PSM: 1.6%
Zapala et al. [31]	OPN	N=46 patients. Endophytic Tumors: 17 patients. Exophytic Tumors: 29 patients. Mean age (years): Endophytic Tumors: 61 years (± 11). Exophytic Tumors: 63 years (± 9). RENAL Nephrometry Score: Not explicitly mentioned, but all tumors were T1a RCC with comparable complexity.	Operative Time: Median 120 minutes for endophytic tumors, longer than 101 minutes for exophytic tumors (P = 0.03). WIT: Median 12 minutes, consistent across both groups. EBL: Similar between groups, not statistically significant. Oncological Outcomes Positive Margins: Endophytic tumors: 6%. Exophytic tumors: 3.4% (P = 0.7). Disease Recurrence: Endophytic tumors: 5.9% (1 patient). Exophytic tumors: 13.8% (4 patients). Survival: No significant differences in recurrence-free survival.
Xu et al. [32]	LPN, OPN	N=229 patients. LPN: 42 patients. OPN: 187 patients. Mean age (years): LPN: 53.2 years (± 14). OPN: 51.5 years (± 13.2). RENAL Nephrometry Score: Not mentioned explicitly in the study.	LPN: Operative Metrics: Operative Time: 154 ± 141 minutes, significantly longer than OPN. EBL: 191 ± 166 mL, lower compared to OPN. Hospital Stay: 8.5 ± 3.1 days, shorter than OPN. Renal Function: While eGFR values were not explicitly mentioned, both approaches were noted to have comparable renal function preservation. LPN demonstrated fewer cases of de novo chronic kidney disease (CKD) than OPN. Oncological Outcomes: No significant differences in recurrence rates or oncological control were reported between LPN and OPN. Negative surgical margins:achieved in all cases, confirming the oncological safety of both procedures. OPN: Operative Metrics: Operative Time: 126 ± 91 minutes, shorter than LPN. EBL: 231 ± 222 mL, higher compared to LPN. Hospital Stay: 9.3 ± 3.8 days, longer than LPN. Renal Function: Comparable to LPN, with no significant differences in eGFR decline or renal functional outcomes. Oncological Outcomes: Similar to LPN, OPN achieved negative surgical margins in all cases and comparable recurrence-free survival.
Wu et al. [33]	RAPN, OPN	N=145 patients. RPN: 51 patients. OPN: 94 patients. Mean age (years): RAPN: Median 52 years (IQR: 46–60). OPN: Median 52 years (IQR: 46–60). RENAL Nephrometry Score: Median DAP score: RPN: 6.5 (IQR: 5–7). OPN: 7 (IQR: 6–8).	RAPN: Operative Metrics: Operative Time: 229 minutes (median), longer than OPN. EBL: 100 mL (median), significantly lower than OPN. Hospital Stay: 9 days (median), shorter than OPN. WIT: 21 minutes, comparable to OPN. Renal Function: Postoperative eGFR decline: 6% (median), comparable to OPN. CKD Upstaging: 29.4%, similar to OPN. Oncological Outcomes: PSM: None reported in either group. Local recurrence: 0.8% in OPN, none in RPN. OPN: Operative Metrics: Operative Time: 182 minutes (median), shorter than RPN. EBL: 200 mL (median), higher than RAPN. Hospital Stay: 11 days (median), longer than RPN. WIT: 20 minutes, comparable to RPN. Renal Function: Postoperative eGFR decline: 8% (median), comparable to RPN. CKD Upstaging: 30.9%, similar to RPN. Oncological Outcomes: PSM: None reported. Local recurrence: 1 case (0.8%).
Oh et al. [34]	RAPN, OPN.	N=200 patients. RAPN: 100 patients. OPN: 100 patients. Mean age (years): RAPN: 54.3 ± 11.5. OPN: 54.6 ± 13.4. RENAL Nephrometry Score: Median score: 7 (IQR: 6–9) for both groups.	RAPN: Operative Metrics: Operative Time: 182.9 minutes, longer than OPN (P < 0.001). WIT: 21.9 minutes, similar to OPN (P = 0.734). EBL: 212 mL, comparable to OPN (P = 0.545). Hospital Stay: 5.4 days, significantly shorter than OPN (P < 0.001). Renal Function: Preoperative eGFR: 78.2 ± 18.4 mL/min/1.73 m². Postoperative eGFR (6 months): 72.3 ± 29.5 mL/min/1.73 m². Percentage eGFR Decline: 7.53%, comparable to 1 OPN. Oncological Outcomes: PSM: None reported in RPN. Local Recurrence: None reported. OPN: Operative Metrics: Operative Time: 138.8 minutes, shorter than RPN (P < 0.001). WIT: 21.2 minutes, similar to RAPN (P = 0.734). EBL: 230 mL, comparable to RAPN (P = 0.545). Hospital Stay: 9.3 days, longer than RPN (P < 0.001). Renal Function: Preoperative eGFR: 76.2 ± 20.9 mL/min/1.73 m². Postoperative eGFR (6 months): 71.5 ± 31.2 mL/min/1.73 m². Percentage eGFR Decline: 6.19%, comparable to RAPN. Oncological Outcomes: PSM: 1 case (1%). Local Recurrence: None reported.
Hankins et al. [35]	RMxPNx	N=54 patients. Mean age: 46 years (range: 20–84). RENAL Nephrometry Score: Not specifically mentioned, but tumors were described as highly complex, often endophytic or associated with hereditary syndromes.	Operative Metrics Operative Time: Mean 385 minutes (6.4 hours). EBL: Mean 1434 mL (range: 250–8500). WIT: Used in 18.5% of cases, with a mean time of 23.3 minutes. Conversions: 11% conversion rate. Reasons for conversion: endophytic lesions, adhesions, and vascular injury. Renal Function Preoperative eGFR: Mean 85.4 mL/min/1.73 m². Postoperative eGFR: Immediate postoperative: 60.8 mL/min/1.73 m², representing a 28.8% decline (P < 0.031). At 3 months: Recovered to 82.3 mL/min/1.73 m², a minimal decline from baseline (3.6%). Oncological Outcomes No Positive Margins: The enucleation technique used for all tumors ensured complete excision. No Recurrences or Metastases: None reported during the study period.
Abdullah et al. [36]	RAPN	N=1,836 patients (806 obese, 1030 non-obese). Mean age: Obese Patients: Median 59 years (IQR: 51–65). Non-Obese Patients: Median 61 years (IQR: 52–69). RENAL Nephrometry Score: Obese Patients: Mean 7.3 (±1.9). Non-Obese Patients: Mean 7.1 (±1.9) (P = 0.003).	Obese Patients (BMI ≥30 kg/m²) Operative Metrics: Operative Time: Median 176 minutes (IQR: 142–212), higher than non-obese patients. EBL: Median 150 mL (IQR: 100–250), higher than non-obese patients. WIT: Median 19 minutes (IQR: 15–24), comparable to non-obese patients. Renal Function: Preoperative eGFR: Median 83.5 mL/min/1.73m². Postoperative eGFR Decline: Median 12.26%, similar to non-obese patients. Oncological Outcomes: PSM: 3.5% in obese patients. Local Recurrence: Not reported. Non-Obese Patients (BMI <30 kg/m²) Operative Metrics: Operative Time: Median 165 minutes (IQR: 135–203), lower than obese patients. EBL: Median 100 mL (IQR: 75–200), lower than obese patients. WIT: Median 19 minutes (IQR: 14–24), similar to obese patients. Renal Function: Preoperative eGFR: Median 83.3 mL/min/1.73m². Postoperative eGFR Decline: Median 11.69%, comparable to obese patients. Oncological Outcomes: PSM: 2.8% in non-obese patients. Local Recurrence: Not reported.
Buffy et al. [37]	RAPN	N=255 patients. Median age: 62 years (IQR: 54–69). PADUA Score: Median 11 (range: 10–13).	Operative Metrics Median Operative Time: 165 minutes (IQR: 120–210). WIT: 18.6 minutes (IQR: 15–23). EBL: Median 150 mL (IQR: 100–250). Oncological Outcomes PSM: 1.9% of patients with malignant histology. Disease Recurrence: Local recurrence: 0.4%. Distant metastases: 0.8% during a median follow-up of 28 months. Renal Function: Postoperative renal function showed slight but clinically insignificant increases in serum creatinine, reflecting effective nephron-sparing surgery.
Abate et al. [38]	RAPN, OPN	N=47 patients. RAPN: 25 patients. OPN: 22 patients. Mean Age (Years) RAPN: 63.2 ± 13.0. OPN: 64.4 ± 11.0. RENAL Nephrometry Score: Median PADUA score: RAPN: 8.4 ± 1.8. OPN: 7.8 ± 1.8.	RAPN: Operative Metrics: Hospital Stay: RAPN patients had significantly shorter stays (median 3.5 days) compared to OPN (median 5 days). Operative Time: RAPN had slightly longer mean operative time (145 ± 25 minutes) compared to OPN (140 ± 28 minutes), but the difference was not statistically significant. EBL: RAPN had lower mean blood loss (120 ± 40 mL) compared to OPN (220 ± 60 mL, P < 0.001). Postoperative Recovery: Pain Management: RAPN patients reported significantly lower pain levels at discharge (VAS score: 3 vs. 6, P < 0.001). Opioid use was significantly reduced in the RAPN group, with fewer patients requiring extended analgesic support. Physical Recovery: RAPN patients demonstrated faster physical recovery as measured by improved physical functioning scores (SF-36) at discharge and 30 days (P = 0.011). Gastrointestinal Symptoms: RAPN patients had fewer issues with postoperative appetite loss and constipation compared to OPN (P = 0.044 and P = 0.045, respectively). QoL: RAPN patients had better overall scores in domains of pain relief and physical recovery at early follow-up intervals (discharge and 30 days).By 90 days, no significant differences were observed in QoL scores between RAPN and OPN, indicating equivalent long-term recovery. Oncological Outcomes: Both RAPN and OPN achieved 100% negative surgical margins.No cases of local recurrence or metastatic progression were reported in either group during the study period. OPN: Operative Metrics: Hospital Stay: Longer compared to RAPN (median: 5.5 days, P < 0.001). EBL: Higher than RAPN (OPN: 240 mL, RAPN: 120 mL, P < 0.002). Operative Time: Comparable to RAPN (mean: 180 ± 25 minutes, P = 0.542). Postoperative Pain and Recovery: Pain Scores: Higher than RAPN at discharge (5.6 vs. 3.5, P = 0.004) and at 30 days (4.0 vs. 2.1, P = 0.012). Physical Recovery: Slower compared to RAPN, with poorer physical functioning and higher gastrointestinal symptom scores at discharge. Oncological Outcomes: Negative Surgical Margins: 100%, equivalent to RAPN. Local Tumor Control: No recurrences during follow-up. Renal Function: Renal function outcomes were equivalent to RAPN, with no significant differences in eGFR at discharge or 90 days postoperative.

Table 5 summarizes complications, follow-up period, and comments comparing RAPN and OPN.

**Table 5:** Summary of included studies RAPN: Robotic-assisted partial nephrectomy, OPN: Open partial nephrectomy, PSM: Positive surgical margin, eGFR: Estimated glomerular filtration rate, LPN: Laparoscopic partial nephrectomy, RALPN: Robotic-assisted laparoscopic partial nephrectomy, IG-PCA: Image-guided percutaneous cryoablation, PLOS: Prolonged length of hospital stay, RMxPNx: Robotic-assisted multiport partial nephrectomy, Off-C: off-clamp, NCDB: National Cancer Database, ICU: Intensive care unit, T1a: Tumor is ≤4 cm, T1b: Tumor is >4 cm but ≤7 cm, rRAPN: Robot-assisted retroperitoneal partial nephrectomy.

Study	Procedure	Complications	Follow-up period (months)	Comments
Love et al. [4]	OPN vs. RAPN	RAPN: 12.1%. Clavien-Dindo Grade ≥III complications: PSM: 3.4%. OPN: 25.2%. Clavien-Dindo Grade ≥III complications: PSM: 10.3%.	Median 18	RAPN has emerged as the preferred approach for nephron-sparing surgery due to its minimally invasive nature, lower complication rates, and superior oncological outcomes. OPN remains a critical option for managing more significant, more complex tumors, underscoring the need for individualized treatment planning.
Wang et al. [5]	RAPN vs. OPN	1) RAPN: 15.8%; Minor Complications (Clavien Grade I–II): 13% (wound pain, mild infections, urine retention, transient fever); Major Complications (Clavien Grade ≥III): Severe bleeding, embolization, rare nephrectomy; Conversion to Open Surgery: 3–5% (due to bleeding/visualization issues); Wound Issues: Minimal (reflects minimally invasive techniques); Transfusion Rate: 6.3%. 2) OPN: 28.9%; Minor Complications (Clavien Grade I–II): 24% (wound infections, urinary leaks, prolonged recovery); Major Complications (Clavien Grade ≥III): Severe bleeding requiring transfusions, renal artery thrombosis, ICU admission; Wound Issues: Higher infection rates, delayed healing, postoperative pain; Transfusion Rate: 10.5%.	Median 49 (RAPN), 52 (OPN)	This study supports robotic-assisted partial nephrectomy (RAPN) as a less invasive alternative to open partial nephrectomy (OPN) for treating complex renal tumors. RAPN offers significant advantages, including reduced blood loss, shorter hospital stays, and fewer complications. The oncological and functional outcomes are comparable for both methods. These findings advocate for the broader adoption of RAPN, particularly in high-volume centres with experienced surgeons.
Xia et al. [6]	RAPN Data from the NCDB (2010–2013).	PLOS: Defined as >3 days. Higher rates in low-volume hospitals. The odds of PLOS are significantly lower in high-volume hospitals (OR: 0.45; P < 0.001). PSM: Higher rates in low-volume hospitals (11.8%). Reduced to 4.6% in very high-volume hospitals. Conversion to Open Surgery: More frequent in low-volume hospitals. Significantly lower odds in high-volume hospitals (OR: 0.47; P < 0.001). No specific intraoperative or postoperative complications were reported in this dataset due to the nature of the NCDB.	Not explicitly mentioned	This study confirms that hospital volume significantly predicts better perioperative and oncological outcomes in RAPN. It advocates for centralizing complex procedures like RAPN to high-volume centres to improve patient care and safety. Future research should focus on identifying specific factors contributing to superior outcomes in high-volume settings and addressing disparities in access to such facilities.
Roaldsen et al. [8]	OPN, RAPN	OPN: 33.3%. Minor Complications (Clavien Grade I–II): 26.7%. Common issues include: Postoperative wound infections. Antibiotics or transfusions are required. Major Complications (Clavien Grade III–IV): 5.3%. Grade IIIb: 4% (e.g., reoperations due to bleeding or complications). Grade IVa: 1.3% (organ failure requiring ICU management). No Grade V Complications (Mortality): Zero deaths recorded. RAPN: 30.1%. Minor Complications (Clavien Grade I–II): 26.0%. Common issues include: Wound infections. Need for antibiotics or transfusions. Major Complications (Clavien Grade III–IV): 4.1%. Grade IIIa: 2.4% (e.g., bleeding requiring radiological intervention). Grade IIIb: 1.6% (reoperations due to complications). Grade IVa: None observed. No Grade V Complications (Mortality): Zero deaths recorded.	Not stated	RAPN demonstrated advantages in hospital stay, blood loss, and surgical precision. It had significantly fewer positive surgical margins than OPN without increasing complication rates. While RAPN required longer operative times, this is expected to improve as surgeon experience grows, as the learning curve suggests. The findings validate RAPN as a safe and effective replacement for OPN in nephron-sparing surgery, particularly in high-volume or experienced centres.
Lee et al. [9]	OPN, RAPN	OPN: 6.0%. Minor Complications (Clavien Grade I–II): Postoperative fever, mild infections, and pain. Major Complications (Clavien Grade ≥III): Grade IIIa: 1 case requiring radiological intervention for hemorrhage. Grade IIIb: 3 cases of reoperations due to significant bleeding or complications. Transfusions: 5 cases due to higher blood loss. Severe Complications (Grade IV): None reported. Mortality (Grade V): None reported. RAPN: 3.0%. Minor Complications (Clavien Grade I–II): Postoperative fever, mild infections, and wound issues. Major Complications (Clavien Grade ≥III): Grade IIIa: 1 case of urine leak managed with percutaneous drainage. Grade IIIb: 1 case requiring reoperation for persistent bleeding. Transfusions: 5 cases, comparable to OPN. Severe Complications (Grade IV): None reported. Mortality (Grade V): None reported.	Not stated	This study demonstrates that RAPN is a feasible and safe alternative to OPN for managing renal tumors ≥4 cm, with distinct benefits in reduced hospital stay and better renal functional outcomes at six months. While RAPN requires longer operative and ischemia times, its minimally invasive nature makes it increasingly favourable for appropriately selected patients.
Yamanoi et al. [14]	RAPN, IG-PCA	Overall Complication Rates: RAPN had an overall complication rate of 8.3%, while IG-PCA showed a slightly higher rate of 10.2%. The difference was insignificant (adjusted OR: 1.24, 95% CI: 0.49−3.15; P = 0.65). Major Complications (Clavien-Dindo Grade III or Higher): Significant complications occurred in 3.7% of RAPN cases versus 5.6% of IG-PCA cases. These differences were also not statistically significant (adjusted OR: 1.50, 95% CI: 0.42−5.46; P = 0.53). Types of Complications for RAPN: Bleeding (Grade IVa) occurred in one case. Other complications included colon injury (Grade IIIa), pneumothorax (Grade IIIa), and ureter injury (Grade IIIa), each reported in one patient. Comparison to IG-PCA: IG-PCA complications included urinary tract infection/abscess (Grade IIIa) in two patients, pneumothorax (Grade IIIa) in two patients, ureteral stricture (Grade IIIa) in one patient, and intestinal injury requiring reoperation (Grade IIIb) in one patient. Renal Function Preservation: There was no significant difference in renal function preservation between RAPN and IG-PCA over a 5-year follow-up, indicating comparable functional outcomes in terms of estimated glomerular filtration rate (eGFR).	Median 12 for functional outcomes	The study highlights that RAPN and IG-PCA are effective nephron-sparing treatments for cT1 RCC, offering comparable oncological and renal functional outcomes. RAPN provides superior local control and remains a viable surgical option, while IG-PCA offers the advantage of a shorter hospital stay and non-surgical management.
Jang et al. [15]	RAPN, LPN	Overall Complication Rates: LPN: 28.9%. RAPN: 20.2% (P = 0.363; not statistically significant). Minor Complications (Clavien Grade I–II): LPN: 26.3%. RAPN: 19.1%. Examples include fever, atelectasis, pleural effusion, lymphatic leak, and ileus. Major Complications (Clavien Grade ≥III): LPN: 1 case of pseudoaneurysm managed with angioembolization. RAPN: 1 case of pseudoaneurysm managed similarly. No Clavien Grade IV (life-threatening) complications in either group. Transfusions: LPN: 10.5%. RAPN: 4.5% (P = 0.239; not significant).	Not stated	The study demonstrates that RAPN offers perioperative outcomes comparable to LPN, with advantages in safety, parenchymal preservation, and surgical precision for complex renal tumors. Although operative and ischemia time differences were not statistically significant, RAPN consistently showed trends favouring minimally invasive benefits. These findings support the expanding role of robotic surgery in managing complex renal masses while emphasizing the importance of surgical expertise for achieving optimal outcomes.
Mahmud et al. [16]	RAPN, LPN, OPN	Transfusion Rate (%): RAPN: 4.5% (4 cases) OPN: 8% (7 cases) LPN: 10.5% (4 cases) Clavien-Dindo Grade ≥III: RAPN: rare OPN: high LPN: few Postoperative Pseudoaneurysm Incidence (%): RAPN:1% (4 cases) OPN: 3.4% (9 cases) LPN: 3.3% (4 cases) RAPN, OPN & LPN: Symptoms of RAPN: 100% gross hematuria, 23% clot retention, and 5.8% hemorrhagic shock. No major intraoperative complications or surgical nephrectomies reported.	Median 12	RAPN is the optimal approach for nephron-sparing surgery, balancing precision, safety, and recovery. OPN, while oncologically effective, is associated with higher blood loss, complication rates, and longer recovery times. LPN demonstrates intermediate outcomes but struggles with vascular control, leading to higher transfusion and complication rates compared to RAPN.
Chu et al. [17]	OPN, RLPN.	RLPN shows lesser complications with regards to abdominal injury over OPN. Clavien-Dindo Grade ≥III: RLPN: 4.62% OPN: 16.92%	Details truncated; specifics unclear	RLPN shows Enhanced recovery of gastrointestinal function after surgery, enhanced vascular exposure, and easier control over OPN. OPN is effective for oncological control but comes with higher complication rates and more significant blood loss. RLPN offers shorter recovery times and intermediate complication rates but requires advanced surgical expertise to manage vascular and hemostatic challenges.
Mastroianni et al. [18]	Off-C RAPN, Off-C OPN	Overall complication rate (%): Off-C RAPN: 3.4% Off-C OPN: 21.7% Clavien-Dindo Grade ≤II (%): Off-C RAPN: 1.3% Off-C OPN: 18.3% Clavien-Dindo Grade ≥III (%): Off-C RAPN: 2.1% Off-C OPN: 3.4% Intraoperative Complications: Off-C RAPN: Rare, minimal vascular issues. Off-C OPN: More frequent vascular challenges.	Median 12	Off-C RAPN emerged as a safer and more efficient alternative to off-C OPN, with reduced hospital stays, lower transfusion rates, and minimal postoperative complications. Both approaches maintained equivalent oncological outcomes, underscoring the reliability of robotic-assisted techniques for nephron-sparing surgery.
Kumar et al. [19]	OPN, RAPN, LPN.	OPN: Out of 15 patients, 10 had no complications. Minor wound problems in three patients were classified as Grade I complications. One patient had a Grade II complication that needed minor medical intervention. One patient had a urine leak, which was a Grade III complication; the medical team managed this with the placement of a stent. There were no Grade IV complications in this study. LPN: Out of 12 patients, ten did not experience any complications. One patient had a Grade I complication, while no Grade II complications were reported. However, one case of a Grade III complication caused by severe intraoperative bleeding required the procedure to be converted to open surgery (OPN). There were no Grade IV complications observed. RAPN: Of the 25 patients, 22 had no complications. Two patients had Grade I complications: one developed pneumothorax, and the other showed transient respiratory symptoms; both were managed conservatively. One patient had a Grade II complication; he did not need any serious intervention. No Grade III or IV complications were reported, thus again underlining RAPN as the safest procedure regarding the lowest complication rates.	Median 12–36	RAPN emerged as the safest and most effective approach for small to moderate renal tumors. It has the lowest complication rates, minimal blood loss, and no positive surgical margins. The slightly longer operative times reflect the technical precision required. LPN showed fewer complications than OPN but challenges in managing vascular control and one case of conversion to OPN. OPN, while effective for complex or large tumors, was associated with higher blood loss, more extended hospital stays, and a more significant complication burden, underscoring its invasiveness.
Prata et al. [20]	RAPN, LPN.	RAPN: The complication rate for RAPN was 11.1%, and all recorded complications were minor (Clavien-Dindo Grade ≤II). These included: Minor Bleeding: Managed conservatively with no need for transfusion or reintervention. Transient Postoperative Symptoms: Mild fever and pain resolved without additional intervention. The medical team reported no significant complications (Clavien-Dindo Grade ≥III), reoperations, or readmissions. The low complication rate reflects the safety and precision of the robotic platform. LPN: The complication rate for LPN was 9.7%, with complications also limited to Clavien-Dindo Grade ≤II. These included: Wound-Related Issues: Mild infections managed with antibiotics or dressing changes. Transient Postoperative Symptoms: Including fever and ileus, treated conservatively.	Median 12	RAPN using the Hugo™ RAS System is a safe and efficient technique for nephron-sparing surgery. Compared to LPN, it offers significant advantages in operative time, hospital stay, and surgical precision. Both approaches achieved comparable oncological and renal functional outcomes.
Masoumi-Rvandi et al. [21]	RALPN, OPN, LPN.	RALPN: 2.4%, the lowest among the three groups. Intraoperative: Minimal bleeding was the most common issue, managed conservatively without significant sequelae. Postoperative Complications: All complications were minor (Clavien-Dindo Grade ≤II), such as transient pain and mild infections. There were no reports of severe complications (Grade ≥ III) or readmissions. OPN: 3.8%, the highest among the groups. Intraoperative Complications: The procedure resulted in significant bleeding in some cases, requiring transfusions and extending the operative time. Postoperative Complications: A higher rate of severe complications (Clavien-Dindo Grade ≥III) was observed. These included infections requiring prolonged antibiotics, urine leaks managed with nephrostomy, and one case requiring reoperation. LPN: 3.6%. Intraoperative Complications: Blood events occurred in a limited number of cases, with one case requiring the surgical team to intervene during the procedure. Postoperative Complications: Most were minor wound infections and mild fever, classified as Grade I–II complications. No Grade IV or V complications were observed, reflecting the technique's safety despite technical challenges.	Median 24	RALPN demonstrated the most favourable perioperative outcomes, with shorter operative times, lower blood loss, fewer complications, and faster recovery, making it a preferred option for nephron-sparing surgery. LPN achieved intermediate outcomes, with the lowest estimated blood loss but slightly higher complication rates, reflecting its technical demands. OPN, while effective for large or complex tumors, showed the highest complication rates and blood loss, emphasizing its invasive nature and longer recovery times.
Kawase et al. [22]	RAPN, LPN	RAPN: Overall Complication Rate: 13%. Minor Complications: Grade II events included infection (2%), ileus (3%), and transient depression (1%). Significant Complications: Rare Grade III complications included pseudoaneurysm rupture (1%) and ventricular fibrillation (1%). LPN: Overall Complication Rate: 11.2%. Minor Complications: Grade II events included infection (2.7%), hemorrhage (1.8%), and liver function deterioration (1.2%). Significant Complications: Rare Grade III complications included necrotic cholecystitis (0.6%) and renal death (0.6%).	46.9 (LPN), 35.5 (RAPN)	The study highlights the advantages of RAPN over LPN in achieving superior perioperative outcomes, higher trifecta rates, and fewer complications. While both approaches preserve renal function and achieve oncological safety, RAPN demonstrates enhanced efficiency and safety, reinforcing its role as the preferred minimally invasive technique for nephron-sparing surgery.
Soisrithong et al. [23]	OPN, RAPN, LPN	RAPN: Grade 0-1 (No or Minor Complications): 27 cases (65.85%). Grade 2 (Moderate): 11 cases (26.83%), which included postoperative infections, hypertension, and manageable ileus. Grade 3a (Severe): 1 case (2.44%), an arteriovenous fistula (AVF) requiring successful embolization. Grade 4b (Life-Threatening): 1 case (2.44%), respiratory failure requiring ventilation. Total Complications: 13 cases, with a complication rate of 34.15%. OPN: Grade 0-1 (No or Minor Complications): 15 cases (83.33%). Grade 2 (Moderate): 1 case (5.56%), likely involving wound infections or manageable postoperative issues. Grade 3a (Severe): 1 case (5.56%) related to a renal AV fistula requiring embolization. Grade 4b (Life-Threatening): 1 case (5.56%) involving cardiac arrest requiring ventilation. Total Complications: 3 cases, with a complication rate of 16.67%. LPN: Grade 0-1 (No or Minor Complications): 10 cases (90.91%). Grade 2 (Moderate): 1 case (9.09%) involving mild infections or localized postoperative bleeding. Grade 3a (Severe): None reported. Grade 4b (Life-Threatening): None reported. In this study, we recorded 1 case with a complication, leading to a complication rate of 9.09%.	Not explicitly mentioned	RAPN demonstrated superior trifecta rates, reduced blood loss, and comparable renal functional preservation, albeit with the most extended operative times. It emerged as the most efficient and precise approach for small renal masses. LPN offered favourable outcomes with low blood loss and minimal complications, making it a viable minimally invasive alternative for appropriately selected patients. OPN, while still relevant for large or complex tumors, showed higher complication rates and longer recovery times, reinforcing its role as a fallback option for challenging cases.
Takahara et al. [24]	RAPN, OPN	RAPN and OPN showed identical rates of severe complications (Clavien-Dindo Grade ≥III) at 5.1%. Transfusion rates were also the same for both procedures at 7.7%. RAPN demonstrated fewer minor complications overall, reflecting its minimally invasive nature and precision.	36	The study highlights RAPN as a safer and more efficient surgical option for nephron-sparing surgery, with shorter ischemia times, lower blood loss, and equivalent oncological and functional outcomes compared to OPN. OPN remains effective but is associated with greater invasiveness and perioperative morbidity, making RAPN the preferred choice for most patients.
Lyu et al. [25]	RAPN	Overall Complication Rate: 12.9%. Grade I (Minor): 9.7%. Grade II (Moderate): 2.7%, including postoperative infections. Grade III–IV (Severe): 0.5%, primarily pseudoaneurysm embolization and one respiratory failure case requiring ventilation.	Median 20.2	The study underlines the versatility and safety of the retroperitoneal approach to robotic partial nephrectomy, reporting favourable surgical and oncological outcomes for various tumour locations and complexities. It shows the feasibility of rRAPN in patients with larger tumors and higher BMI, which goes against the traditional contraindications. While complications were rare, the study affirms the importance of surgical expertise in minimizing risks. This large cohort supports rRAPN as a reliable option for nephron-sparing surgery.
Yu et al. [26]	OPN, LPN	Overall Complication Rates LPN: 22.2% OPN: 27.5% There was no statistically significant difference in complication rates between the two approaches. LPN: Minor Complications (Clavien-Dindo Grade I–II): Wound infections and transient ileus were the most common minor complications. These were managed conservatively with antibiotics or supportive care. Major Complications (Clavien-Dindo Grade III–V): Grade III (Severe): A small percentage (2.5%) of patients experienced urinary leaks requiring intervention such as ureteral stenting or nephrostomy. Hematomas requiring radiological drainage were rare. Grade IV–V (Life-Threatening): No Grade IV or V complications were reported for LPN, reflecting its safety for appropriate candidates. OPN: Minor Complications (Clavien-Dindo Grade I–II): Unlike LPN, wound infections and transient ileus were the most common minor complications. The incidence of minor infections was slightly higher in OPN due to the invasive nature of the procedure. Major Complications (Clavien-Dindo Grade III–V): Grade III (Severe): Urinary leaks occurred in 3.1% of patients, requiring stenting or percutaneous drainage. Postoperative hematomas and prolonged fever needing intervention were also observed but were rare. Grade IV (Life-Threatening): One patient experienced respiratory failure necessitating ventilatory support. Grade V (Fatal): No deaths were reported for OPN during the study period.	Not explicitly mentioned	The study demonstrates that laparoscopic partial nephrectomy (LPN) serves as a viable minimally invasive alternative to open partial nephrectomy (OPN) for nephron-sparing surgery, particularly for low-complexity renal tumors. LPN offers benefits such as reduced blood loss, shorter hospital stays, and comparable oncological outcomes. Although surgeons need more operative and clamp time for moderate-complexity tumours with LPN, its safety and efficacy remain on par with OPN. When selecting a procedure, it is essential to consider tumor complexity, the surgeon's expertise, and specific patient factors.
Xu et al. [27]	RAPN using the KD-SR-01 robotic system	Overall Complication Rate: 29.4%. Clavien-Dindo Grade I Complications: Three patients required antiemetics for postoperative nausea. Two patients required analgesics for pain management. No Major Complications: We did not encounter any complications classified as Grade III or higher and did not need to perform any reoperations or blood transfusions.	Median 6 (short-term)	This study again confirms the feasibility and safety of the KD-SR-01 Robotic System for nephron-sparing surgery. The results are favourable regarding low complications, excellent preservation of renal function, and negative surgical margins, heralding this as a new, good alternative to already available robotic platforms like da Vinci. The sample size is small; however, these findings suggest that KD-SR-01 has significant potential as a low-cost solution for robotic surgery.
Seyam et al. [28]	RAPN	Overall Complication Rate: 18.8%. Minor complications (Clavien-Dindo Grade I–II) included transient fever, wound infections, and mild postoperative ileus, which accounted for the majority of cases. Major Complications (Clavien-Dindo Grade III-IV): One arteriovenous fistula requires embolization. One case of atrial fibrillation requires intensive care unit admission. The conversion rate to open surgery is 8.9%, primarily because of bleeding or technical difficulties.	Median 30	This study emphasizes the safety and feasibility of RAPN in a developing country setting, with results comparable to those obtained from a larger international series. The slightly higher conversion rate reflects the learning curve of adopting RAPN in lower-volume centres. Overall, the outcomes confirm RAPN as a reliable approach for nephron-sparing surgery, with excellent oncological and functional results.
Boylu et al. [29]	RAPN, OPN	Overall Complication Rate: RAPN: 17.3%. OPN: 20%. Minor Complications (Grade I–II): RAPN: Sub-ileus (2 cases), mild wound infections (2 cases), and transient creatinine elevation (1 case). OPN: Wound infections (2 cases), blood transfusions (2 cases), and prolonged drainage (1 case). Major Complications (Grade III-IV): None reported for either group.	Median 36	The study demonstrates that RAPN offers a minimally invasive alternative to OPN, with advantages in terms of blood loss, hospital stay, and patient recovery. Although RAPN requires longer operative and ischemia times, the overall complication rate was slightly lower than OPN, and renal functional and oncological outcomes were equivalent. This supports RAPN as a safe and effective option for nephron-sparing surgery, particularly for small renal masses.
Kim et al. [30]	RAPN, OPN	OPN: Overall complications: 28.1%. Major complications (Clavien Grade III–V): 18.8%. Common issues: Wound dehiscence, bleeding, and pseudoaneurysms, pneumonia. RAPN: Overall complications: 21.2%. Major complications (Clavien Grade III–V): 11.8%. Common issues: Pneumothorax, ileus, and urinary retention.	Median 30	The study shows that robotic-assisted partial nephrectomy (RAPN) yields outcomes similar to open partial nephrectomy (OPN) for highly complex renal tumors. It also has the benefits of shorter hospital stays and fewer major complications. These results highlight the feasibility of robotic-assisted surgery for complex cases, mainly when performed in high-volume centres by experienced surgeons.
Zapala et al. [31]	OPN	Endophytic Tumors: Overall Complication Rate: 5.9%. Minor complications included wound infections and ileus (Clavien Grade II). No severe complications (Grade ≥III). Exophytic Tumors: Overall Complication Rate: 10.3%. Minor complications included transient ischemic attack and hypokalemia. One severe complication requiring reoperation for bleeding (Grade III).	Median 47	Researchers support the safety and efficacy of OPN for treating endophytic tumors, as they face technical challenges that are likely to reduce success rates compared to exophytic tumors. The series once again demonstrated that complete intraparenchymal renal masses may be safely and effectively treated by NSS, confirming skilled surgical techniques and intraoperative tools as determining factors in good results.
Xu et al. [32]	LPN, OPN	LPN: Overall Complication Rate: 35.71%. Minor Complications (Clavien-Dindo Grade I–II): Gastrointestinal Issues:Constipation: 2.38% of patients experienced mild constipation postoperatively. Stress Ulcers: 4.76%, requiring proton pump inhibitors (PPIs) for management. Minor Gastrointestinal Bleeding: 2.38%, controlled conservatively. Other Minor Events: Mild urinary retention resolved with catheterization—one case of transient ileus. Major Complications (Clavien-Dindo Grade III-IV): No Grade III or IV complications were reported for LPN, indicating its safety. OPN: Overall Complication Rate: 36.36% of patients experienced complications, including both low-grade and high-grade complications​. Low-Grade Complications (Grades I and II): Most complications fall under this category. High-Grade Complications (Grades III and above): A smaller proportion of the complications were severe.	Median 12	Laparoscopic nephrectomy is a viable alternative to open nephrectomy, offering less blood loss, shorter stays, and fewer wound complications. It requires skilled surgeons for optimal outcomes.
Wu et al. [33]	RAPN, OPN	Overall Complication Rate: RAPN: 25.5%. OPN: 18.1% (not statistically significant, P = 0.293). Minor Complications (Grade I–II): RAPN: 23.5%, including delayed bleeding, mild urinary leaks, and fever. OPN: 17%, with mild urinary leaks and transient ileus. Major Complications (Grade III-IV): RAPN: 2%, one case of severe bleeding requiring intervention. OPN: 1.1%, one case of reoperation for bleeding.	Median 12	RAPN is a safe, effective alternative to OPN, with less blood loss, shorter stays, and faster recovery. Both methods have similar outcomes, but RAPN has slightly more minor complications, supporting its use for less complex renal tumors.
Oh et al. [34]	RAPN, OPN.	Overall Complication Rate: RAPN: 10%. OPN: 8%. Minor Complications (Grade I–II): RAPN: 4 cases of prolonged hematuria and 2 cases of wound infections. OPN: 3 cases of pleural injury and 2 cases of urinary leakage. Major Complications (Grade III-IV): RAPN: 1 case of prolonged bleeding requiring angioembolization. OPN: 3 cases requiring angioembolization for prolonged bleeding.	Median 24	This matched-cohort study confirms that RAPN provides equivalent oncological and renal outcomes to OPN, with several additional advantages, such as reduced hospital stay and postoperative analgesic usage. The marginally prolonged operative time for RAPN is traded off by its minimal invasiveness, thereby reaffirming RAPN as an attractive alternative to OPN in managing small renal masses. Further prospective studies are recommended to authenticate the same.
Hankins et al. [35]	RMxPNx	Overall Rate: 11%. Major Complications: Conversions to open surgery: 6 cases (11%). Endophytic lesions: 3 cases (5.5%). Adhesions: 2 cases (3.7%). Vascular injury leading to radical nephrectomy: 1 case (1.8%). Minor Complications: Not explicitly mentioned, but no Clavien Grade ≥III complications beyond conversions.	Median 3 (Short-term)	This study demonstrates the feasibility and safety of RMxPNx for managing multiple renal tumors in one kidney, achieving excellent preservation of renal function and excellent oncologic outcomes despite a highly complex caseload. It underscores again that such a challenging operation needs advanced surgical skills and is linked to very tight patient selection criteria. Robotic multiplex partial nephrectomy presents a minimally invasive alternative to conventional open surgery, especially for patients harbouring hereditary renal cancer syndromes or displaying multifocal disease.
Abdullah et al. [36]	RAPN	Overall Rate (Clavien Grade ≥III): Obese Patients: 4.4%. Non-Obese Patients: 3.5%. Common Complications: Intraoperative hemorrhage, urine leaks, and minor infections. Rare enterotomy and mesenteric hematoma, resolved without sequelae.	Median 18	This multicenter study validates the safety and efficacy of RAPN in obese patients. Obesity was associated with slightly longer operative times and higher blood loss but did not significantly impact complication rates or oncological and renal functional outcomes. These findings highlight RPN as a feasible and practical approach for managing renal masses in obese patients, supporting its use in high-volume centres with experienced surgeons.
Buffi et al. [37]	RAPN	Overall Complication Rate: Clavien-Dindo Grade I–II (Minor): 24.3%. The symptoms include mild hematuria, fever, and minor infections.Clavien-Dindo Grade III–V (Major): 5.1%. Major complications included: Embolization was performed in four cases to address bleeding. JJ stent placement for urinary leakage (5 cases). Two cases required reintervention due to surgical complications. Conversion Rates: The conversion rate to radical nephrectomy is 1.9% due to bleeding or the inability to achieve negative margins.	Median 28	This multicenter study confirms the safety and efficacy of RAPN in managing highly complex renal tumors. Despite tumor complexity, RAPN accomplished excellent oncological outcomes with low complications and practical renal preservation. It underlines the importance of surgeon expertise and advanced intraoperative technologies for optimizing outcomes in complex renal masses. Further studies are needed on such advanced techniques' long-term oncologic impact and economic feasibility.
Abate et al. [38]	RAPN, OPN	RAPN : (Overall Complication Rate: 12%. Minor bleeding (2 cases). Transient ileus requiring conservative management (1 case). OPN: (Overall Complication Rate: 14%.Postoperative infections requiring antibiotics (3 cases). Pain requiring prolonged opioid therapy (4 cases).)	Median 3	This study provides strong evidence supporting RAPN as the preferred approach for small renal masses, particularly in the early postoperative period, due to better pain management, shorter recovery, and comparable oncological outcomes. The findings reinforce the minimally invasive advantages of RAPN while demonstrating no significant differences in long-term quality of life between the two methods. Further research is recommended to evaluate the long-term oncological outcomes and cost-effectiveness of RAPN.

Analysis of Overall Complication Rates of OPN Across the Studies

OPN complication rates vary widely in different studies, from 3.4% to 36.36% [4,5,8,9,16-19,21,23,24,26,29-34,38]. Complex tumor cases or older surgical methods lead to higher complication rates (e.g., 36.36% in Xu et al. [32]). On the other hand, better surgical skills and careful patient selection result in lower rates (e.g., 3.4% in Mahmud et al. [16]).

**Figure 2:**
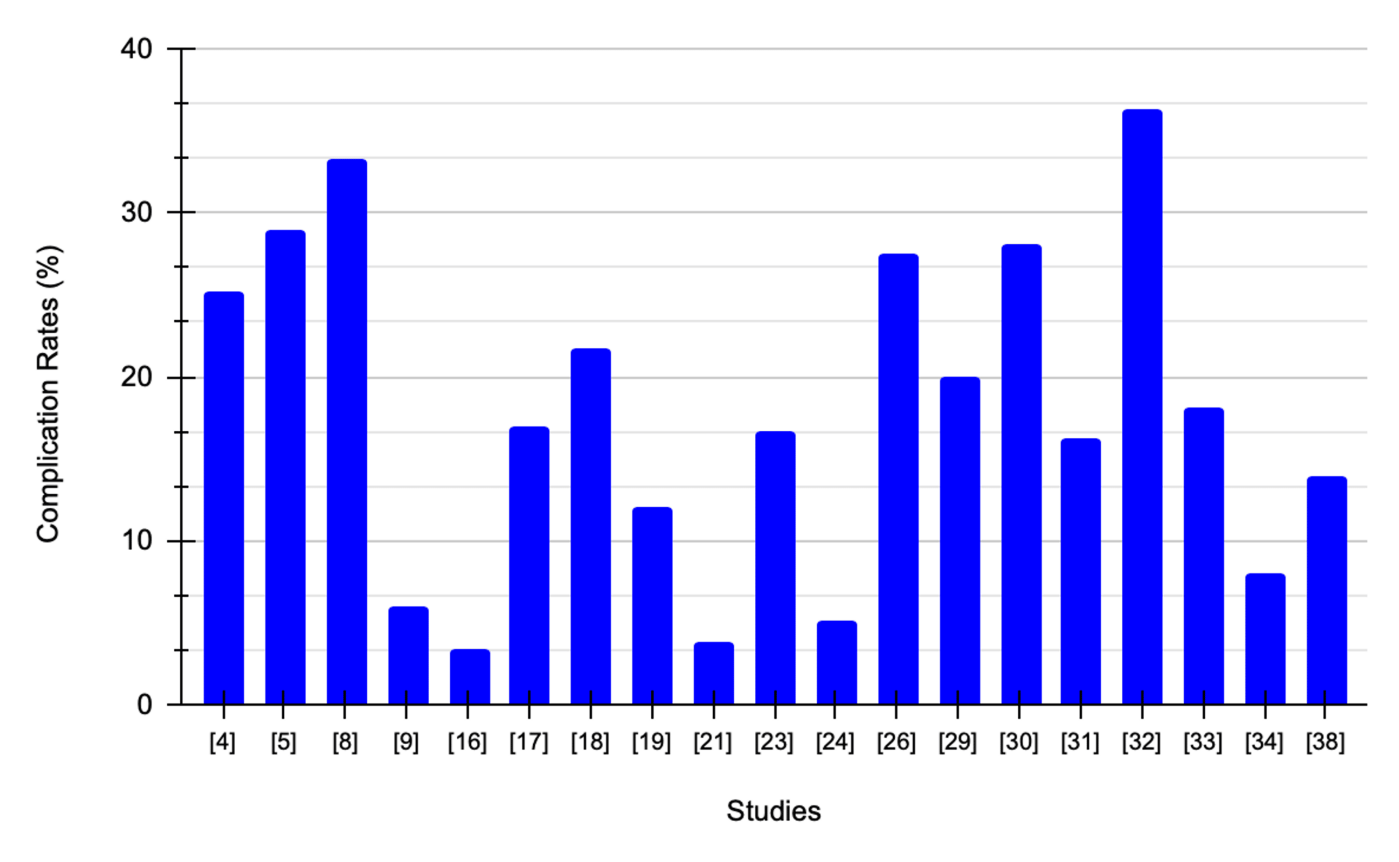
Overall complication rate (%) for OPN across studies OPN: Open partial nephrectomy.

Most studies report mid-range complication rates (15%-28%) [17,18,23,26,29,30,31,33]. Wang et al. (28.9%) and Boylu et al. (20%) are examples of this trend [5,29]. These numbers show that OPNs are reliable when done by experienced surgeons, but open surgery still has risks. The variations in rates thus reflect that factors like patient complexity, the volume of surgeries, and the hospital's expertise come into significant play in outcomes. That would suggest that while OPN is reliable, it might be less effective in complication reduction than other more invasive techniques such as RAPN. Figure 2 presents the overall complication rate for OPN.

Analysis of the Overall Complication Rates of RAPN Across the Studies

**Figure 3:**
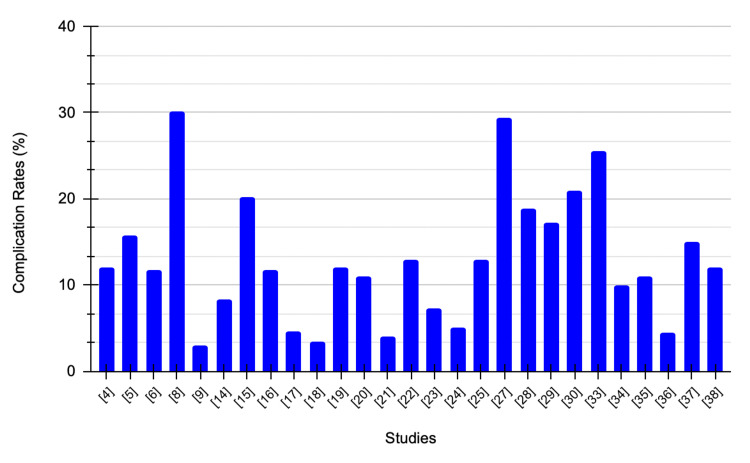
Overall complication rate (%) for RAPN across the studies RAPN: Robotic-assisted partial nephrectomy.

The overall complication rates for every study using RAPN differ significantly across the board, going by the ranges of tumor complexity and institutional and surgeon expertise, from 3% to 30.10% [4-6,8,9,14-25,27-30,33-38]. The reports of low rates of complications, like 3% from Lee et al. and 4% from Masoumi-Rvandi et al., are attributed to perfection in patient selection and organisational capability within the study [9,21]. These more optimal conditions have moderate rates, including 12.1% from Love et al. and Buffi et al., with 15% [4,37]. Studies with higher complication rates, such as 30.10% from Roaldsen et al. and 25.50% from Wu et al. pertain to more advanced stages or the study of centres with a smaller sample size [8,33]. These increased rates indicate how much more intricate the tumor or the institution’s learning curve became. Regardless of the numerous advantages of OPN, there is consistency in the ability of RAPN to be more favorable in the mode of complication events. The visual illustration of the overall complication rate (%) for RAPN is shown in Figure 3.

Understanding Complication Rate Averages for RAPN and OPN

**Figure 4:**
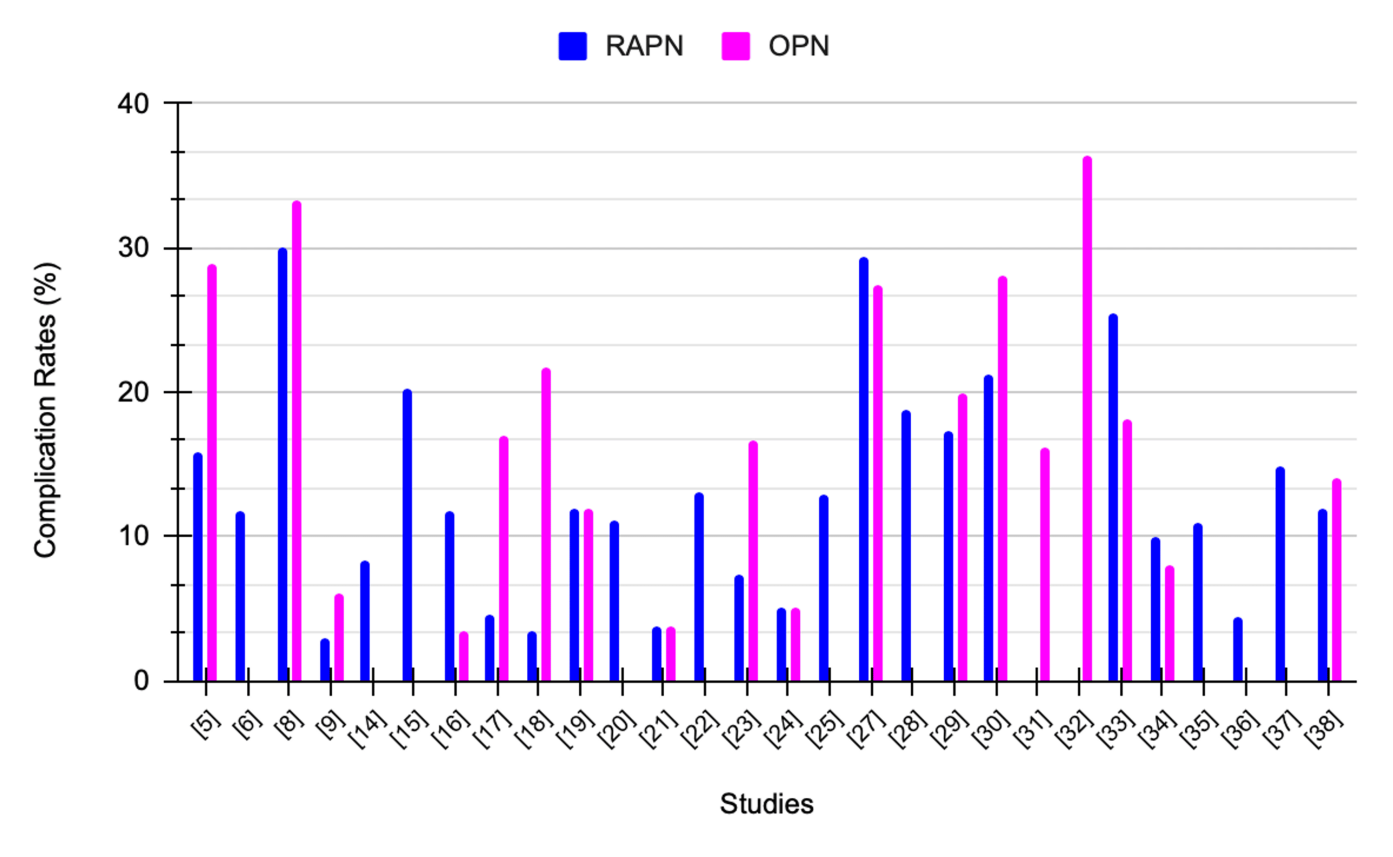
Comparison of complication rates (%) between RAPN and OPN across multiple studies, highlighting the overall lower mean complication rate for RAPN (13%) compared to OPN (18%) OPN: Open partial nephrectomy, RAPN: Robotic-assisted partial nephrectomy.

RAPN has 13% means of complication rates [4-6,8,9,14-25,27-30,33-38]. In comparison, 18% indicates that for OPN [4,5,8,9,16-19,21,23,24,26,29-34,38]. We can conclude that RAPN is superior in reducing complications, ostensibly due to its less traumatic and more accurate technique. OPN’s higher percentage paints a picture where there is more operative trauma from the open techniques, leading one to deduce that RAPN is the preferable method whenever robotic machines and skills are at their disposal. Figure 4 shows the graphical comparison of the rapport of complications between RAPN and OPN.

Interpretation of Negative Surgical Margin (NSM) Rate Averages for RAPN and OPN

**Figure 5:**
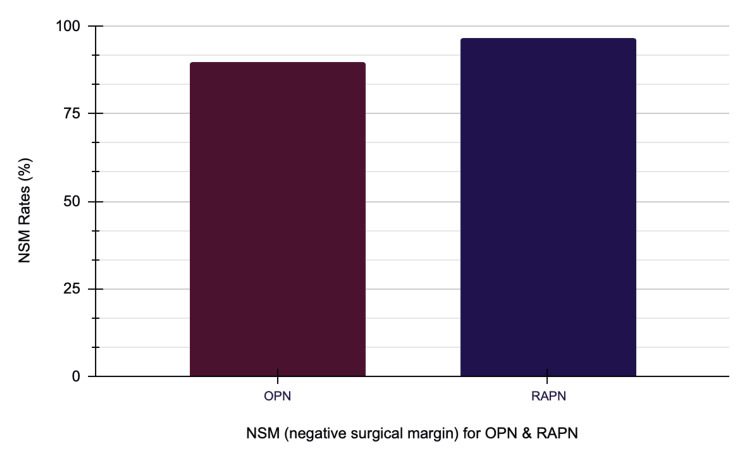
Comparison of NSM rates (%) between RAPN and OPN across multiple studies, highlighting the overall lower mean complication rate for RAPN (97.16%) compared to OPN (92%) NSM: Negative surgical margin, OPN: Open partial nephrectomy, RAPN: Robotic-assisted partial nephrectomy.

The comparison of NSM rates across the included studies demonstrates the oncological reliability of both OPN and RAPN. On average, OPN achieved an NSM rate of 92% [4,8,9,14,18,21,23,24,29,30,33,34,36,38], while RAPN consistently performed better with an average NSM rate of 97.16% [4,8,9,14,18-21,23-25,27-30,33,34,36-38]. RAPN's superior precision in tumor removal was evident in studies such as [4,8,18], which reported significantly higher NSM rates for RAPN compared to OPN (96.6% vs. 89.7%, 95.8% vs. 78.4%, and 96.6% vs. 87.8%, respectively). Although some studies have shown that OPN can achieve remarkable outcomes, with NSM rates reaching 100% in some instances [9,23], this is primarily seen when performed by skilled surgeons. Even so, the NSM rates that are more often than not linked to RAPN show how effective it is in controlling residual disease burden, especially in complex situations and in high-volume medical centers. There is an essential caveat as several investigations combining RAPN and OPN did not report NSM rates [5,6,15,16,17,22,26,31,32,35]. Several studies for OPN did not report on NSM rates [19,20,25,27,28,37]. This has left some void in the evaluation and analysis, which points to inconsistencies in future reporting requirements. In the end, the persistence of the NSM rates above the norm retrospectively stamped RAPN's character as a method of choice for nephron-sparing surgery, averaging precision and accuracy. Figure 5 presents a graph of NSM rates for RAPN and OPN.

Discussion

This systematic review compiles findings from 30 studies comparing RAPN and OPN. This discussion will explore key findings related to perioperative outcomes, functional preservation, oncological safety, and the impact of patient selection in the context of RAPN and OPN; it is essential to understand their differences in perioperative outcomes. 

RAPN proves to be the superior option, demonstrating several benefits. Patients who undergo RAPN generally experience shorter hospital stays, averaging three days, compared to six days for those who have OPN [18,34,38]. Additionally, RAPN is associated with significantly less mean EBL, measuring 181 mL compared to 284 mL for OPN, as noted by several studies [5,8,9,16,17,19,21,23,24,29,30,33,34,38]. These findings highlight the benefits of RAPN in reduced hospital stays. Some other considerable benefits after RAPN include shorter recovery times, low blood transfusion rates, and fewer instances of wound infection and severe bleeds, according to the studies by Wang et al. [5] and Roaldsen et al. [8]. Although RAPN takes longer, with an average operative time of 173 minutes compared to 149 minutes for OPN, the duration reflects the complexity of the robotic procedures, as noted by Lee et al. [9]. Jang et al. mentioned that RAPN and LPN effectively minimize pseudo-aneurysms, occurring in only 1% of cases [15]. However, Mahmud et al. encountered postoperative pseudo-aneurysms more with OPN, 3.4% cases, LPN with 3.3% cases, and rare cases with RAPN [16].

Mastroianni et al. and Soisrithong et al. demonstrated RAPN's effectiveness in complex cases, attaining trifecta achievements and lower transfusion rates than OPN [18,23]. Kumar et al. and Prata et al. highlighted RAPN's advantages in reducing blood loss and hospital stay, particularly in high-volume centers [19,20]. Masoumi-Ravandi et al. emphasized that RAPN reduces overall EBL compared to OPN, improving perioperative and postoperative outcomes (Clavien-Dindo Grade ≤II). They reported transient pain and mild infections, while they did not observe any severe complications (Grade ≥III) or readmissions [21].

Oncological Outcomes

Love et al. and Wang et al. reported similar RFS rates and CSS rates following RAPN as compared to OPN [4,5]. These findings further support the oncological equivalence of both techniques and reassure us about the effectiveness of each method. Zapala et al. pointed out that OPN effectively manages tumors with high PADUA scores, emphasizing its crucial role in complex anatomical challenging tumors [31]. On the other hand, Buffi et al. illustrated that RAPN successfully achieves trifecta outcomes, characterized by a positive margin rate of 1.9%, ischemia times not exceeding 25 minutes, and an absence of significant complications [37]. Xu et al. supported RAPN's oncological precision, maintaining a 100% NSM using the KD-SR-01 robotic system [27].

Functional Outcomes

RAPN consistently preserved renal function more effectively than OPN. Hankins et al. and Kawase et al. reported smaller declines in eGFR among patients undergoing RAPN [35,22]. Kim et al. highlighted RAPN's nephron-sparing advantage due to shorter ischemic times, which minimized renal injury [30]. Furthermore, Wu et al. and Oh et al. emphasized the requirement of angioembolization due to severe bleeding more in OPN than in RAPN [33,34]. 

Complications

Complications remain critical in comparing RAPN and OPN, with notable differences in frequency and severity observed across the 30 studies. The types of complications were predominantly classified using the Clavien-Dindo system, ranging from minor complications (Grade I-II) to major complications (Grade III-IV). RAPN consistently demonstrated a lower overall complication rate, with reduced instances of high-grade complications.

Across the studies, RAPN showed a mean complication rate of 13% [4-6,8,9,14-25,27-30,33-38], significantly lower than the 18% reported for OPN [4,5,8,9,16-19,21,23,24,26,29-34,38]. High-grade complications (Clavien-Dindo Grade III or higher) were more common in OPN, as highlighted in studies such as Kim et al. and Roaldsen et al. which reported high overall complication rates of 18.8% and 33.3%, respectively, for OPN [34,8]. In contrast, RAPN reported significant complications at a reduced rate, typically below 15% in most studies, as seen in Masoumi-Rvandi et al. and Lee et al., with rates as low as 3%-4% [21,9]. Leilei Xia et al. reported that improved dexterity and visualization with the robotic platform contributed to superior vascular control, leading to less EBL and improved perioperative outcomes compared to OPN [6].

Mahmud et al. and Mastroianni et al. reported that RAPN leads to lower transfusion rates [16,18]. In contrast, Mastroianni et al. reported OPN showing higher rates of intraoperative bleeding along with a higher incidence of wound infections, vascular challenges, and extended hospital stays [18].

Boyle et al. found that patients who underwent OPN experienced wound infections and prolonged drainage lasting more than six days, while patients who underwent RAPN had no such issues [29]. Kim et al. reported lower rates of postoperative pulmonary infections and overall infection rates in RAPN patients compared to OPN​ [30]. Oh et al. reported that 2% of OPN cases resulted in urine leakage that required stenting. Still, the RAPN group had no such cases. This finding suggests that the precise technique used in renorrhaphy during RAPN reduces the risk of these leaks [34].

RAPN offers advantages over OPN, including reduced postoperative pain and a faster recovery period. According to Abate et al., one of the key benefits of RAPN is its ability to lessen postoperative discomfort and decrease the length of hospital stays [38]. Furthermore, many studies demonstrate that RAPN is linked to a lower incidence of severe complications (Grade III or higher). Surgeons frequently associate high-grade complications in OPN with increased surgical trauma, prolonged ischemic times, and delayed recovery. Kim et al. focused explicitly on highly complex tumors. They found comparable complication rates (Clavien 3-5) between RAPN (11.8%) and OPN (18.8%), indicating that RAPN was capable of managing anatomically challenging cases without a compromise in outcomes​ [30]. Wu et al. also presented similar complication rates for RAPN and OPN despite performing more frequent RAPN for larger or more complex tumors [33].

The complication rates for RAPN and OPN varied significantly depending on institutional expertise and surgeon experience. Studies by Abdullah et al. and Buffi et al. emphasized that high-volume centers consistently reported better outcomes with RAPN, even in complex cases. Conversely, OPN was associated with higher complication rates in low-volume centers, particularly for anatomically challenging tumors [36,37].

Patient-Centered Outcomes

Boylu et al. and Kim et al. emphasized RAPN's benefits in improving patient-reported outcomes, including quicker rehabilitation and better postoperative satisfaction [29,30]. This focus on patient-centered outcomes is crucial for understanding the holistic impact of RAPN. Xu et al. and Buffi et al. reiterated the importance of RAPN in achieving long-term functional and oncological success, particularly for complex renal masses [32,37].

Clinical Implications

RAPN offers a superior perioperative profile with reduced morbidity, lower blood loss, shorter hospital stays, and oncological precision. These advantages establish RAPN as the promising approach in high-resource centers with adequate infrastructure and expertise. Its ability to manage complex renal tumors, especially those with high PADUA or RENAL scores, reinforces its importance in advanced nephron-sparing surgery.

The financial and training demands of RAPN require a strategic approach to its implementation. It is essential to prioritize this procedure for patients most likely to benefit from complex tumors. By adopting this targeted approach, we can ensure that the resources allocated to RAPN yield the best possible outcomes.

OPN remains vital in resource-limited settings, especially for surgeons who do not have access to robot-assisted systems or expertise. As open procedures may have higher rates of perioperative and postoperative complications like wound infections, they can be an appropriate choice for less complex cases.

These findings emphasize the necessity for tailored treatment strategies that consider institutional capabilities, patient needs, and tumor complexity based on the Clavien-Dindo Grade. By doing this, RAPN and OPN can remain relevant in clinical practice, ensuring that patients receive the most suitable and effective care tailored to their unique circumstances.

Strengths and Limitations

The review stresses many strengths of the thirty studies that were included. The studies include multiple measures such as perioperative, postoperative, oncological, functional and patient-centred measures. Such a broad scope allows for an appreciation of the impact of various surgical methods. Moreover, the data emerges from both straightforward cases and those involving very complex renal tumors, thus making the results more generalizable.

The data quality is impressive since all studies were assessed and rated high. Furthermore, measures include patient-centered characteristics of the analysis, which are frequently overlooked when comparing procedure modalities, such as quality of life after surgery. Similarly, applying standard measures like the Clavien-Dindo classification for complications ensures that the comparisons of the studies are valid and reliable throughout many studies.

Nevertheless, the studies included also have some significant weaknesses. Since most of the studies were observational, which had limitations in determining the cause-and-effect relationship. Different types of study designs, including retrospective cohort studies and diverse institutional practices, can lead to biases that impact the outcomes reported. Numerous studies had to classify their findings according to the complexity of the tumors or the experience of the surgeons involved. This dependence on classifications-particularly for more challenging procedures or less experienced surgeons-often resulted in insufficiently differentiated outcomes.

In addition, the lack of consistent reporting on long-term oncological and functional results across various studies hinders a comprehensive understanding of the advantages of RAPN. The authors do not frequently mention one or several limitations - availability of resources, especially access to robotic systems - which may reduce the applicability of the findings.

Future Directions

RAPN and OPN should be systematically compared and contrasted in further studies, as this will aid standardization and improve clinical decision-making. Further and intense studies are essential in determining CSS rates, maintaining healthy renal function and quality of life across various communities, and selecting the complexities of the tumor. It is necessary to recognize that assessing arguments through common sense suggests that without performing a cost-effectiveness analysis - especially in low-resource settings - one cannot fully understand the economic implications of RAPN and its clinical benefits.

The effectiveness of RAPN concerning hilar or deep endophytic lesions and highly complex tumors remains unclear. This calls for more RCTs. Secondly, RAPN promotes health equity by making use of available infrastructure as well as enhancing scalability. Robotics and augmented reality also facilitate better outcomes and quicker learning for RAPN, and their integration into the surgery is plausible. Future studies should thus include RCTs. Outlining denormalized and standardized training regulations and competency benchmarks is vital since those variables guarantee uniform results. Finally, high-quality multicenter trials are needed to inform updates to clinical guidelines, ensuring the evidence-based integration of RAPN and OPN for personalized and optimal patient care.

## Conclusions

OPN and RAPN are key methods used in kidney-sparing procedures, but they differ significantly in how they are performed and yield results. OPN is often the better choice in settings with limited resources, as it can provide effective cancer control and preserve kidney function when conducted by skilled surgeons. However, it is usually associated with a higher rate of perioperative complications.

Conversely, RAPN brings advanced robotic technology to the same oncology performance, with increased accuracy and reduced ischemia time. This technique has proved very effective, especially in minimizing blood loss and recovery. However, the substantial resources required for RAPN infrastructure and training may limit access and widespread adoption, particularly for complex kidney tumors. RAPN has become more common in advanced medical centers. Further research is therefore needed to fill the gaps in the comparative evaluation of this technique with OPN. Future research on the long-term outcomes and cost-effectiveness of RAPN for treating complex tumors is a focal point. Creating standardized training protocols and strengthening access to robotic technology will help develop clinical guidelines for individualized disease management to ensure equitable care.
